# Mystifying Molecular Structure, Expression and Repertoire Diversity of IgM Heavy Chain Genes (*Ighμ)* in *Clarias* Catfish and Hybrids: Two Novel Transcripts in Vertebrates

**DOI:** 10.3389/fimmu.2022.884434

**Published:** 2022-06-17

**Authors:** Anurak Bunnoy, Uthairat Na-Nakorn, Prapansak Srisapoome

**Affiliations:** ^1^ Laboratory of Aquatic Animal Health Management, Department of Aquaculture, Faculty of Fisheries, Kasetsart University, Bangkok, Thailand; ^2^ Center of Excellence in Aquatic Animal Health Management, Faculty of Fisheries, Kasetsart University, Bangkok, Thailand; ^3^ Laboratory of Aquatic Animal Genetics, Department of Aquaculture, Faculty of Fisheries, Kasetsart University, Bangkok, Thailand; ^4^ Academy of Science, The Royal Society of Thailand, Bangkok, Thailand

**Keywords:** *Clarias* catfish, IgM, novel transcript, characterization, structural analysis

## Abstract

Two novel immunoglobulin heavy chain (*Ighμ*) transcripts encoding membrane-bound forms of IgM (mIgM) were discovered in bighead catfish*, Clarias macrocephalus*. The first transcript contains four constant and two transmembrane domains [Cμ1-Cμ2-Cμ3-Cμ4-TM1-TM2] that have never been reported in teleosts, and the second transcript is an unusual mIgM that has never been identified in any vertebrate [Cμ1-(Cδ2-Cδ3-Cδ4-Cδ5)-Cμ2-Cμ3-TM1-TM2]. Fluorescence *in situ* hybridization (FISH) in bighead catfish, North African catfish (*C. gariepinus*) and hybrid catfish revealed a single copy of *Ighμ* in individual parent catfish, while two gene copies were found in diploid hybrid catfish. Intensive sequence analysis demonstrated multiple distinct structural variabilities in the VH domain in *Clarias*, and hybrid catfish were defined and used to generate diversity with various mechanisms. Expression analysis of *Ighμ* in *Aeromonas hydrophila* infection of the head kidney, peripheral blood leukocytes and spleen revealed significantly higher levels in North African catfish and hybrid catfish than in bighead catfish.

## Introduction

Vertebrate immunoglobulins (Igs) are the hallmark elements in adaptive immune responses to a particular antigen with high discrimination, specificity and long-term memory. Igs are generated by B cells and serve two purposes: 1) cell-surface receptors (membrane-bound forms; mIgs) for signaling and activation of cells and 2) soluble effector molecules (secreted forms; sIgs) for neutralization of microbes and toxins, opsonization (immunophagocytosis), antibody-dependent cell-mediated cytotoxicity (ADCC), and complement activation (immunolysis) (1).

The basic structure of Ig heavy chain (*Igh*) gene molecules consists of variable (VH) and constant (CH) regions (2). The gene sequence organizations of *Igh* vary depending on the species (3, 4). The immunologic effector functions of the Ig classes are determined by the different constant regions of the CH chain. In most mammals, five classes of Igs are categorized based on different gene sequences; these classes are μ, δ, γ, ϵ, and α, and they correspond to the five major isotypes of Igs, IgM, D, G, E, and A, respectively. In teleost fish, only three *Igh* isotypes have been identified, namely, IgM, D, and T/Z based on the gene sequences of the isotypes μ, δ, and τ/ζ, respectively (5–7). Additionally, distinct *Igh* classes have been identified in nonmammalian vertebrates, including IgNAR and IgW (in cartilaginous fish) (8), IgO (platypus), IgP (in *Pleurodeles waltl*) (9), IgX, IgF and IgY (in amphibians) (9, 10).

The evolution of Ig molecules in jawed vertebrates diverged from the “multicluster” type to the “translocon” type approximately 470 million years ago (11, 12). The *Igh* translocon configuration in jawed vertebrates consists of variable (V_H_), diversity (D_H_), joining (J_H_), and constant (C_H_) regions, although the organization of these regions varies depending on different species, such as mouse, V_H_-D_H_-J_H_-Cμ-Cδ-Cγ3-Cγ1-Cγ2b-Cγ2a-Cε-Cα; human, V_H_-D_H_-J_H_-Cμ-Cδ-Cγ3-Cγ1-ψε-Cα1Cγ2-Cγ4-Cε-Cα2; rabbit, V_H_-D_H_-J_H_-Cμ-Cγ-Cε-Cα-Cα (13 Cα genes were repeated); and cattle, V_H_-D_H_-J_H_-Cμ-Cδ-Cγ3-Cγ1-Cγ2-Cε-Cα4. In teleosts, gene organization appears to be an intermediate type between the multicluster and translocon types in its evolution from Chondrichthyes to tetrapods. The *Igh* genes possess a translocon configuration, V_H_-D_H_-J_H_-Cζ/τ-(V_H_)-D_H_-J_H_-Cμ-Cδ, similar to those of tetrapods, although there are differences in the Cζ/τ gene locations among teleost species and other vertebrate groups (13, 14). Interestingly, in catfish groups such as channel catfish (*Ictalurus punctatus*), the Cζ/τ genes are not found either in the 3’-region of the VH gene cluster or within the VH gene (15).

The *Ighμ* gene was the first Ig class identified in teleosts and has long been considered the most primitive and most prevalent Ig in fish plasma. It can be expressed as mIg or sIg. Secreted tetrameric IgM represents the main Ig in the serum of teleosts. In teleosts as well as a wide range of mammalian species, molecular characterization of the *Ighμ* gene has revealed that the secreted *Ighμ* transcripts consist of a rearranged VDJ region spliced to the Cμ_1_ domain, followed by Cμ_2_, Cμ_3_ or Cμ_4_ (VH-Cμ_1_-Cμ_2_-Cμ_3_- Cμ_4_). Interestingly, the unusual transcript patterns of the *Ighμ* gene indicate that the membrane *Ighμ* transcripts in teleost fish appear to be shorter than those in mammals since the first transmembrane (TM) exon is spliced directly to Cμ_1_, Cμ_2_ or Cμ_3_ thereby excluding the entire Cμ_2_ or/and Cμ_3_ or/and Cμ_4_ exons (VH-Cμ_1_-TM, or VH-Cμ_1_-Cμ_2_-TM, or VH-Cμ_1_-Cμ_2_-Cμ_3_-TM, respectively). These patterns have been identified and reported in many teleost species (3, 16–27).

In Thailand, the production of catfish is the second largest fish aquaculture industry, with approximately 159,314 million tons in 2004 (28). Two major species of catfish, bighead catfish (*Clarias macrocephalus* Gunther, 1864) and North African catfish (*C. gariepinus*), and their hybrid catfish (“pla-duk-big-uey” in Thai), are economically popular cultured species in southeast Asia, especially in Thailand, Vietnam, Malaysia and Indonesia. Notably, the hybrid catfish (*C. macrocephalus* × *C. gariepinus*) demonstrates many commercially desirable dominant characteristics from its parents, such as good quality of meat, rapid growth, better feed conversion, increased survival, resistance to many diseases and tolerance to many environmental conditions. Therefore, bighead catfish has become a remarkable supporter species in catfish production in many counties in southeast Asia.

Most of our current knowledge on the immune systems of catfish comes from other models and economically important fish species, such as zebrafish (*Danio rerio*) and medaka (*Oryzias latipes*) (14, 29–35). In addition, the structural, functional and genetic features of catfish Igs in the genus *Clarias* are still virtually absent.

The study of molecular *Ig* genes in catfish could provide a better understanding of their role in immunity, and these genes could be applied as tools for basic research, diagnosis, and therapy in catfish culture farming. Thus far, the study of *Ig* genes has focused on the molecular structure of *Ighμ* loci in catfish, particularly their splicing patterns, functions, diversification, expression and homogeneity or heterogeneity between species and their hybrids. In the present study, the organization of the *Ighμ* genes was analyzed at the chromosomal level. The obtained data provide new knowledge regarding catfish and may benefit applications in sustainable aquaculture industries.

## Materials and Methods

### Animals

Bighead catfish (*Clarias macrocephalus* Günther, 1864), North African catfish (*Clarias gariepinus*) and their hybrid catfish (*C. macrocephalus* × *C. gariepinus*), 90–120 g in body weight, were obtained from the Department of Aquaculture, Faculty of Fisheries, Kasetsart University, Thailand. The fish were acclimatized in a quarantine tank with aerated freshwater at temperatures between 28 and 31°C for 2 weeks before the start of the experiment. The experimental procedures performed with aquatic animals were carried out in accordance with the Ethical Principles and Guidelines for the Use of Animals National Research Council of Thailand for the care and use of animals for scientific purposes. The protocol was approved by the Animal Ethics Committee, Kasetsart University, Thailand (Ethics ID: ACKU61-FIS-004).

### Isolation of Genomic DNA and RNA and cDNA Synthesis

Catfish genomic (g) DNA was isolated from whole blood tissues using a QIAamp DNA Blood and Tissue Mini Kit (QIAamp, CA, USA) according to the manufacturer’s protocols.

The PBLs of catfish are target tissues used to isolate total RNA and for cDNA synthesis. Total RNA was analyzed using NucleoZOL™ reagent (Clontech Laboratories, CA, USA) according to the manufacturer’s instructions. The obtained total RNA was then used as templates for first strand cDNA synthesis using the protocol described for the Thermo Scientific RevertAid Reverse Transcriptase Kit (Thermo Fisher Scientific, MA, USA). The products of the gDNAs and first-strand cDNA synthesis were stored at -80°C for further experiments.

### Amplification of the Internal Constant Domain of cDNA Encoding *Ighμ* Genes

Degenerative primers were first designed to amplify the internal constant domain region of cDNA encoding *Ighμ* genes based on the highly conserved regions of *Ighμ* genes from the NCBI nucleotide database of closely related species of catfish, including *Ictalurus punctatus* (M27230), *Hemibagrus macropterus* (JF909893), *Silurus meridionalis* (KJ659069) and *Pelteobagrus fulvidraco* (JN202623). The primers were *Cla*_*Ighμ_intr_f:* 5′-GTYTMCMSYDTGGCARTGCGGCBC-3′ and *Cla*_*Ighμ_intr_r:* 5′-GARVYCTCTGGTGGAGSGAGCAMG*-*3′ with an approximate amplicon size of 950 bp. Reverse transcription PCRs (RT-PCRs) were carried out using Phusion High-Fidelity DNA Polymerase (Thermo Fisher Scientific, Waltham, MA, USA) according to the manufacturer’s protocols. The PCR cycling conditions are one cycle of 95°C for 5 min, 30 cycles of 95°C for 30 sec, 55°C for 30 sec, and 72°C for 90 sec, followed by a final extension at 72°C for 5 min. Then, PCR products encoding *Ighμ* genes were ligated to the pJET1.2/blunt cloning vector (Thermo Fisher Scientific, Waltham, MA, USA) according to the manufacturer’s protocols. Nucleotide sequencing of the recombinant plasmid was performed by the Macrogen sequencing service (Macrogen Inc., Seoul, South Korea) using pJET1.2 forward and reverse sequencing primers.

### Recovery of 5′- and 3′-Constant Domains of cDNA Encoding *Ighμ* Genes

The 5′- and 3′- internal constant domain sequences of cDNA encoding *Ighμ* genes were recovered by rapid amplification of cDNA ends (RACE) PCR techniques using 5′- and 3′-SMARTer^®^ (Clontech, Mountain View, CA, USA), according to the manufacturer’s protocol, with SMARTer^®^ universal primers and gene-specific primers that were provided from the kits and designed from 5′- or 3′- internal constant domain sequences, respectively (*Cla*_*Ighμ_5RACE_r:* 5′-GTGCCGCTCGCATCCTTCCAAACG-3′ and *Cla*_*Ighμ_3RACE_f:* 5′-GGCTCAACTTCTCCAGTTAAGTG-3′). The 5′ and 3′ RACE-PCR products were cloned into the pJET1.2/blunt cloning vector (Thermo Fisher Scientific, Waltham, MA, USA) and sequenced using the Macrogen sequencing service (Macrogen, Inc., Seoul, South Korea) as described above.

### Cloning and Characterization of *Ighμ* Genes of Catfish

To obtain and characterize the *Ighμ* genes of catfish, we used catfish gDNA to amplify the *Ighμ* genes of catfish using forward and reverse specific primers designed from the highly conserved region of the 5′ end of Cμ_1_ and the 3′UTR of full-length *Ighμ* cDNAs, respectively. The forward primer was *Cla*_*Ighμ_Cμ_1__f:* 5′-GAACGTCGGTGACCGTAACTTCA-3**′** for all *Clarias* catfish species. The reverse primers were *Cla*_*mac*_*Ighμ_3UTR_r:* 5**′-**GAACACACAAGCATCAGACAGACTG*-*3**′** for big head catfish and hybrid catfish and *Cla*_*gar*_*Ighμ_3UTR_r:* 5**′-**CAAGAACACACAAGCATCAGACAG*-*3′ for North African catfish and hybrid catfish.

PCRs were carried out using Phusion High-Fidelity DNA Polymerase (Thermo Fisher Scientific, Waltham, MA, USA) according to the manufacturer’s protocols. The PCR cycling conditions were one cycle of 95°C for 5 min, 30 cycles of 95°C for 30 sec, 60°C for 30 sec, and 72°C for 5 min, followed by a final extension at 72°C for 10 min. The PCR products of *Ighμ* genes were cloned and sequenced as described above.

### Sequence and Phylogenic Analyses of Full-Length cDNAs and *Ighμ* Gene

The obtained nucleotide sequences of *Ighμ* genes were characterized, analyzed, assembled, and aligned using bioinformatic software DNA sequences including GENETYX version 7.0, BLASTN (https://blast.ncbi.nlm.nih.gov/), BLASTX (https://blast.ncbi.nlm.nih.gov/), ExPASy (https://www.expasy.org/), DAS-Transmembrane Prediction server (www.sbc.su.se/~miklos/DAS/), MatGAT program version 2.0 (http://www.bitincka.com/ledion/matgat), and MEGA version 7.0 (http://www.megasoftware.net). Furthermore, the sequences were aligned with those of related *Ighμ* gene sequences in other vertebrate species using ClustalW. Phylogenetic trees were constructed using Molecular Evolutionary Genetics Analysis (MEGA) software, version 7.0 (Proprietary freeware, Japan) with neighbor-joining (NJ) algorithms with a bootstrap of 1000 replications.

### Prediction of Protein Structures of Catfish *Ighμ* Genes

The protein structures of *Ighμ* molecules of catfish were determined to predict the possibility of constant domain structure using the structural bioinformatics web server SWISS-MODEL workspace integrates programs (https://swissmodel.expasy.org) according to the programs’ procedures (36). The amino acids in the constant domain of each *Ighμ* molecule were used to generate the target sequence for protein structure prediction.

### Cytogenetic Mapping of *Ighμ* Genes in Catfish

Metaphase chromosomes of catfish were obtained from the head kidney of each catfish species treated with colchicine according to Karami et al. (2015) (37). Prepared tissues were stored in fixative solution at -20°C until use. The chromosomal spreads were analyzed by staining with 15% Giemsa for 45 min and examined under a light microscope (Olympus, MA, USA). The *Ighμ* gene probes for fluorescence *in situ* hybridization (FISH) used full-length *Ighμ* genes containing the constant domain Cμ_1_ to the 3′UTR of the *Ighμ* genes of each catfish species. DNA probes were performed according to standard procedures of FISH Tag detection kits (Thermo Fisher Scientific, MA, USA). Metaphase chromosome hybridization was carried out following a previously described method (38). Chromosomes and specific probes were performed with DAPI and Alexa Fluor 488 or 594 dyes following the manufacturer’s instructions (Thermo Fisher Scientific, MA, USA). Digital micrographs were recorded and processed using a Nikon/DigitaL Eclipse C2Si confocal microscope (Nikon Instruments Inc., NY, USA).

### Diversity Analysis of the Variable Domain of *Ighμ* Genes of Catfish

The 5′ VH region of first-strand cDNAs encoding *Ighμ* genes was synthesized from total RNA of PBLs of each catfish species using a 5′ RACE-Ready cDNA synthesis kit (Clontech, Mountain View, CA, USA), as previously described. PCR was amplified using Phusion High-Fidelity DNA Polymerase (Thermo Fisher Scientific, Waltham, MA, USA) with the 5′ universal primer mix (UPM) and gene-specific primers (GSPs) from the kit and nucleotide sequences located in the 5′ region of the Cμ_1_ constant domain of *Ighμ* genes (*Cla_Ighμ_Cμ1_r*: 5′-GTGCCGCTCGCATCCTTCCAAACG-3′). The PCR cycling conditions were one cycle of 95°C for 5 min, 25 cycles of 95°C for 30 sec, 68°C for 30 sec, and 72°C for 120 sec, followed by a final extension at 72°C for 5 min. The 5′ RACE VH PCR products of *Ighμ* cDNA were ligated to the pJET1.2/blunt cloning vector (Thermo Fisher Scientific, Waltham, MA, USA) according to the manufacturer’s protocol. A different hundred recombinant clones were randomly selected for sequencing by the Macrogen sequencing service (Macrogen Inc., Seoul, South Korea) as previously described.

### Analysis of V_H_, D_H_ and J_H_ Segments

The 5′ VH nucleotide sequences of *Ighμ* genes were trimmed to three separated regions encoding the *V_H_*, *D_H_* and *J_H_* genes using an Ig variable domain sequence analysis tool (NCBI IgBLAST, https://www.ncbi.nlm.nih.gov/igblast/). The nucleotide sequences of each gene region were classified at the family level based on 80% similarity of nucleotide sequences using the matrix global alignment tool (MATGAT 2.0).

### Analysis of Complementarity-Determining Regions (CDRs)

CDR nucleotide regions of each variable domain nucleotide sequence were first characterized using IgBLAST (https://www.ncbi.nlm.nih.gov/igblast/). The D_H_ segments were predicted using the VDJsolver 1.0 server (https://services.healthtech.dtu.dk). The CDRs were analyzed for their characterization, including CDR length distribution and amino acid composition in each position, using the High V-QUEST IMGT tool (39).

### Analysis of the Variability Plot of the Variable Domains of IgM Molecules

The variability plots of variable domain nucleotide sequences were analyzed using the Shannon (1948) (40) and Kabat and Wu (1971) (41) methods. The variability plots were illustrated using the online bioinformatics software protein variability server (PVS, https://imed.med.ucm.es/PVS/pvs-help.html).

### Expression of *Ighμ* Genes in Catfish

The tissue distribution of *Ighμ* genes of catfish was addressed in both normal fish and fish that underwent *Aeromonas hydrophila* challenge. Fifty acclimatized catfish were randomly transferred into tanks containing 500 L of water. Catfish were used to determine the expression of the *Ighμ* gene in normal fish and infectious fish. The infectious catfish were intraperitoneally injected with 0.1 mL of virulent *A. hydrophila* AQH0018 bacterium (1 × 10^6^ CFU/mL). A virulent *A. hydrophila* AQH0018 bacterium was grown in TSB medium at 32°C for 18 hr, and cell pellets were harvested by centrifugation at 2,500 rpm for 10 mins, washed and resuspended in 0.85% NaCl prior to injection. The effective doses were preliminarily determined to validate the optimum dose throughout the median lethal dose (LD_50_) assay (42). Sixteen organs, brain (BR), dendrite (DR), gall bladder (GB), gills (GIL), head kidney (HK), heart (HR), intestine (IN), liver (LI), muscle (MC), ovary (OV), peripheral blood lymphocytes (PBL), skin (SKN), spleen (SPL), stomach (STO), testes (TES) and trunk kidney (TK), were collected from three fish of each catfish before injection (normal fish) and post injection (infectious fish) at 0, 12, 24, 36, 48, 60, 72, 84, 96, 108, 120, 132, 144, 156 and 168 hr.

Total RNA and first-strand cDNA synthesis from sixteen organs of catfish were isolated using NucleoZOL™ reagent (Clontech Laboratories, CA, USA) and a Thermo Scientific RevertAid Reverse Transcriptase kit (Thermo Fisher Scientific, MA, USA) following the manufacturer’s instructions.

Quantification of *Ighμ* gene expression was performed using quantitative reverse transcriptase PCR (qRT-PCR) assays. qRT-PCR assays were performed with Brilliant III Ultra-Fast SYBR^®^ Green (Agilent, CA, USA) in Mx3005P QPCR Systems (Agilent, CA, USA). qPCRs of the *Ighμ* gene and *β-actin* genes of each fish species were conducted using the specific primers *Cla*_*Ighμ_qpcr___f:* 5′-TGGACTGAGCTACGTTTGGAAGGA-3′ and *Cla*_ *Ighμ_ qpcr_r:* 5′-CGCCTGACTCACTGAGGAGTACTT*-*3′ with an amplicon size of 167 bp and *Cla*_*b-actin_qpcr___f:* 5′-GTCCGTGACATCAAGGAGAAGCTC-3′ and *Cla*_*b-actin_qpcr___r:* 5′-GGACTCCATACCCAGGAAAGATGG-3′ with an amplicon size of 189 bp for the *Ighμ* and *β-actin* genes, respectively. In addition, the expression distribution of two novel transcripts including *Cm*_mIgM2 and *Cm*_mIgM3 were performed in sixteen organs of healthy bighead catfish. The specific primers are *Cm*_mIgM2_*f:* 5′-GCACAGAGTTCACCTGCAATG-3′ and *Cm*_mIgM2_*r:* 5′-AAATGTTAAGGCATACAGACC-3′ with an amplicon size of 154 bp, and *Cm*_mIgM3_*f:* 5′-CCACGGATACGTCTGGAGAA-3′ and *Cm*_mIgM3_*r:* 5′-ACTCGCCTTGACTCTCACTG-3′ with an amplicon size of 163 bp.

qRT-PCR cycling conditions were one cycle of 95°C for 5 min, 40 cycles of 95°C for 30 sec, 60°C for 30 sec, and 72°C for 90 sec, followed by a final extension at 72°C for 10 min. The expression of the *β-actin* gene was used as the housekeeping gene to standardize the results by eliminating variation in mRNA and cDNA quantity and quality. The relative expression of the *Ighμ* gene in catfish tissues was calculated using 2*^-ΔΔCT^* analysis according to the protocol by Schmittgen and Livak (2001) (43). The brain RT was used as calibrator. All reactions were done in triplicate.

### Statistical Analysis

The relative expression levels of *Ighμ* genes were calculated using *β-actin* as a reference. All quantitative data are presented as the mean **±** standard deviation (SD). Statistical analysis at each time course was performed using SPSS statistics 24.0 software using one-way analysis of variance (ANOVA) and Duncan’s new multiple range test (DMRT). The level of statistical significance between species in different organs is indicated as * (*P*<0.05), ** (*P*<0.01) and *** (*P*<0.001) using Student’s t-test.

## Results

### Characterization and Sequence Analysis of cDNA Transcripts Encoding a Constant Region of the *Ighμ* Gene

**Figure 1:**
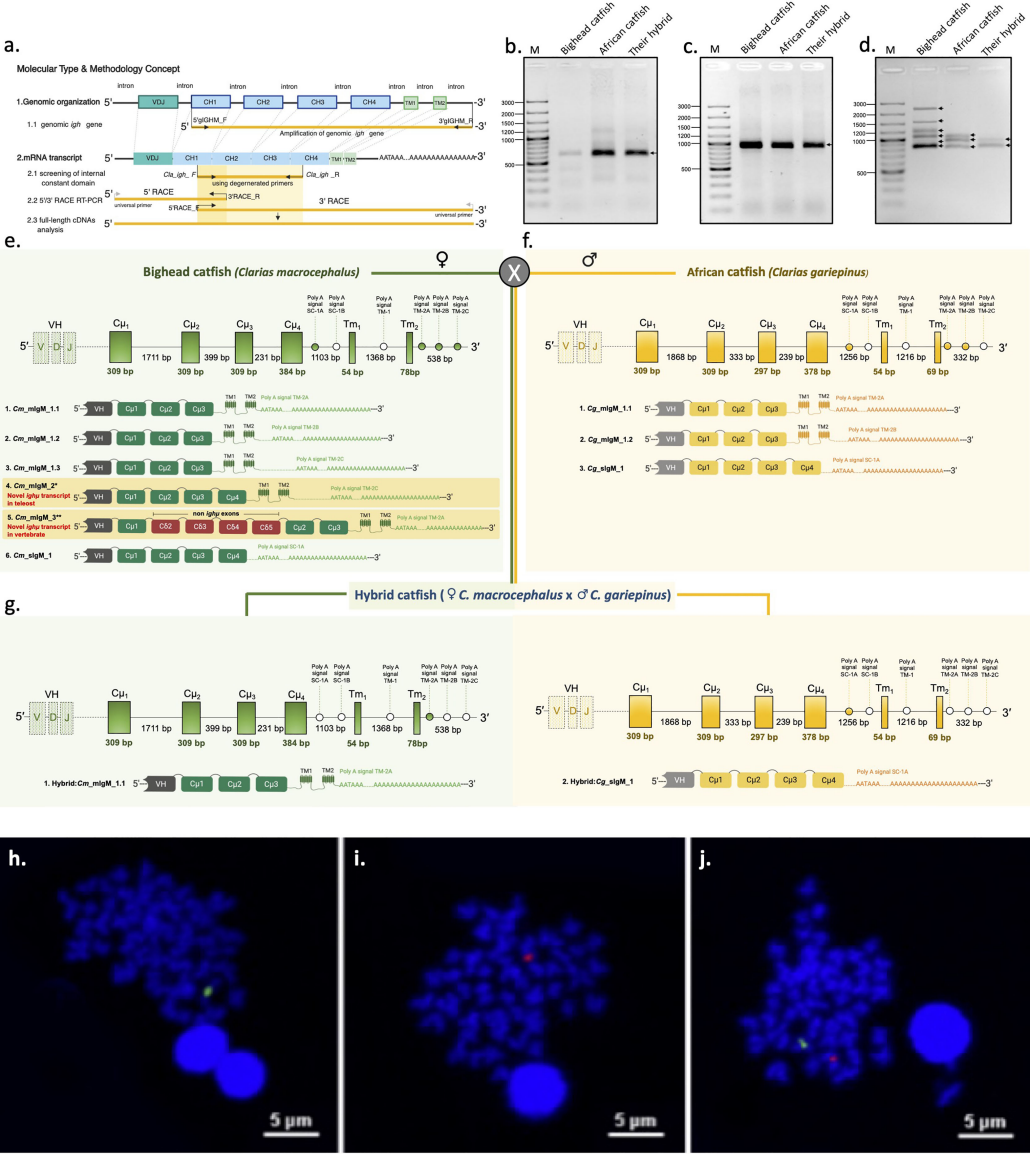
Representation of the genomic organization and splicing patterns of *Ighμ* in teleosts and catfish. *Ighμ* generally encodes a VH domain, four constant (Cμ_1_ to Cμ_4_) domains and two transmembrane domains. For full-length cDNA *Ighμ* transcripts in bighead catfish, North African catfish and their hybrid catfish, three major molecular techniques were performed **(A)**. Patterns of PCR amplification products, 5’ RACE PCR **(B)**, internal constant domain **(C)**, and 3’ RACE PCR **(D)** of *Ighμ* of bighead catfish, North African catfish and their hybrid catfish. A single gene copy (NCBI accession no. MZ559374) and six different *Ighμ* transcripts were identified in bighead catfish, and five and one were expressed as membrane (NCBI accession nos. MN934742, MN934743, MN934744, MN934745 and MN934746) and secreted forms (NCBI accession no. MN934747), respectively. Moreover, two membrane forms of *Ighμ* transcripts had novel splicing patterns in teleosts and vertebrates (highlighted in yellow) (NCBI accession nos. MN934745 and MN934746) **(E)**. A single gene copy (NCBI accession no. MZ559375) and three different *Ighμ* transcripts were identified in North African catfish; two and one were expressed as membrane (NCBI accession no. MN934748 and MN934749) and secreted forms (NCBI accession no. MN934750), respectively **(F)**. Two gene copies (NCBI accession nos. MZ559376 and MZ559377) and two *Ighμ* transcripts were identified in the hybrid catfish (NCBI accession nos. MN934751 and MN934752) **(G)**. Comparisons of cytogenetic localization of *Ighμ* gene loci in bighead catfish **(H)**, North African catfish **(I)** and their hybrid catfish **(J)**. All nucleotide sequences are illustrated in supplementary files and the NCBI database corresponding to the provided NCBI accession numbers.

Through analysis of the cDNA transcripts encoding constant regions of the *Ighμ* genes in bighead catfish, North African catfish and their hybrid catfish, one hundred positive clones of each catfish were first cloned and sequenced to obtain the internal nucleotide sequences of the constant regions of the *Ighμ* genes. A single band of PCR fragment was shown for each catfish species. Sequence analysis indicated no variation in the nucleotide sequences of individual bighead catfish, North African catfish and their hybrid catfish. There were four exons encoding Cμ_s_ [Cμ_1(partial)_-Cμ_2_-Cμ_3_-Cμ_4(partial)_] of 954 and 949 bp for bighead catfish and North African catfish, respectively (**Figure 1A**), compared to the published *Ighμ* sequence of channel catfish (*Ictalurus punctatus*). In the hybrid, two different nucleotide sequences were observed; each sequence exhibited a 100% match with the *Ighμ* sequence of one of the parents. Among a hundred clones from hybrid catfish, the *Ighμ* nucleotide sequences shared a distribution of 28% and 72% to bighead catfish and North African catfish, respectively (**Figure 1A**).

The 5′ and 3′ of the *Ighμ* gene constant regions in each catfish were consequently cloned and sequenced using 5′ and 3′ RACE techniques with specific primers designed from the 5′ end of the Cμ_2_ exon and 3′ end of Cμ_1_ for recovering the 5′ and 3′ nucleotide regions of *Ighμ* cDNAs, respectively. The obtained 5′ and 3′ nucleotide sequences of *Ighμ* genes corresponded to the Cμ_1_-Cμ_2_-Cμ_3_ and Cμ_3_-Cμ_4_-TM-polyA tails, respectively (**Figure 1A**). No variation difference in the nucleotide sequences was observed in the 5′ *Ighμ* constant region exon in bighead catfish and North African catfish. In addition, the hybrid catfish exhibited two different nucleotide sequences, one from each parent (**Figures 1A, B**). There was no variation in the internal nucleotide sequences of the constant regions of the Ighμ genes in all studied catfish. (**Figure 1C**). However, the sequences of the 3′ constant regions consisted of two major different Ig forms, (mIg and sIg), which are controlled by alternate mRNA processing events in certain species. Up to six nucleotide sequence patterns of 3′ region exons were observed in bighead catfish (**Figure 1D**): five patterns for mIg and one pattern for sIg. Among the five patterns of mIg, three corresponded to Cμ_1_-Cμ_2_-Cμ_3_-TM1-TM2 with differences in the polyadenylation signal region (polyA signal: TM-2A, TM-2B and TM-2C, respectively) of the untranslated 3′ sequence, one corresponded to Cμ_1_-Cμ_2_-Cμ_3_-Cμ_4_-TM1-TM2 containing the polyA signal TM-2C, and one corresponded to Cμ_1_-(non-*Ighμ* exons)-Cμ_2_-Cμ_3_-TM_1_-TM_2_ containing the polyA signal TM-2A. One sIg was encoded by exon Cμ_1_-Cμ_2_-Cμ_3_-Cμ_4_ and the polyA signal SC-1A (**Figures 1D, E** and **Supplementary Figures 1**–**6**).

Three nucleotide sequence patterns were observed in North African catfish, and they corresponded to two patterns for mIg and one pattern for sIg (**Figure 1D**). Two patterns of mIg corresponded to Cμ_1_-Cμ_2_-Cμ_3_-TM1-TM2 with differences in the polyadenylation signal region (polyA signal: TM-2A and TM-2B, respectively) of the untranslated 3′ sequence. Another pattern corresponded to sIg that contained four exons coding Cμ_1_-Cμ_2_-Cμ_3_-Cμ_4_ and the polyA signal SC-1A (**Figures 1D, F** and **Supplementary Figures 7**–**9**).

Two nucleotide sequence patterns of the 3′ region exon corresponding to mIg (Cμ_1_-Cμ_2_-Cμ_3_-TM1-TM2) and sIg (Cμ_1_-Cμ_2_-Cμ_3_-Cμ_4_) were observed in the hybrid. The mIg and sIg nucleotide sequence coding from Cμ_1_ through polyA tails were absolutely identical to their parent, bighead catfish and African catfish, respectively (**Figures 1D, G** and **Supplementary Figures 1**, **9**).

### Cytogenetic Mapping of *Ighμ* Genes in Catfish

*In situ* hybridization with specific probes to the *Ighμ* gene of individual catfish species, bighead catfish and North African catfish revealed that only a single copy of the *Ighμ* gene was found in bighead catfish (**Figure 1H**) and North African catfish (**Figure 1I**). In addition, a strong hybridization of both the *Ighμ* genes of bighead catfish and North African catfish was represented in their hybrid catfish (**Figure 1J**). The expression of *Ighμ* transcripts in the hybrid catfish from the two *Ighμ* genes received from the parents was confirmed.

### Amino Acid Sequence Comparison of *Ighμ* Transcripts in *Clarias* Catfish

**Figure 2:**
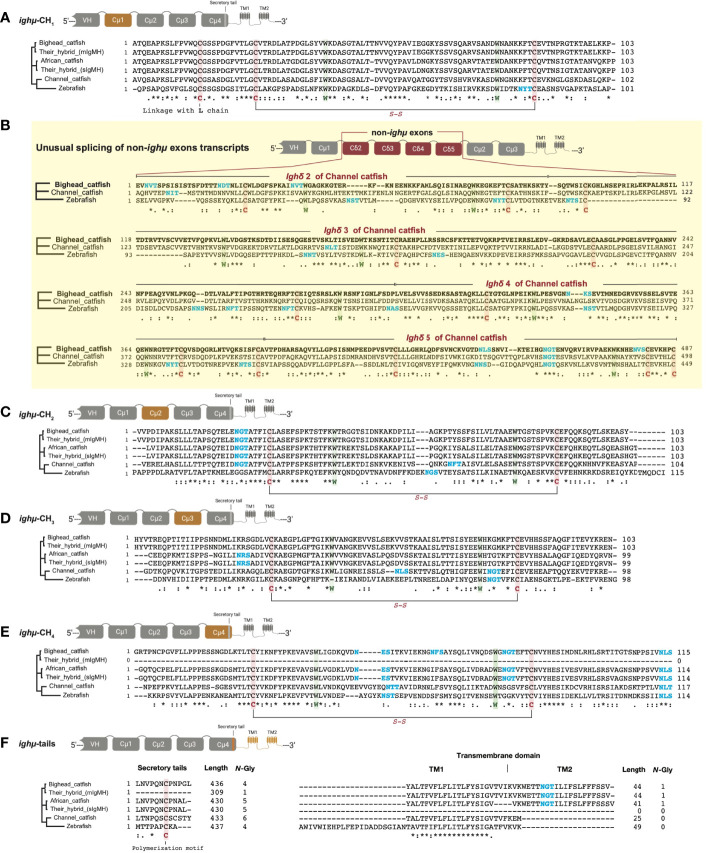
Comparisons of translated amino acids among bighead catfish, North African catfish, their hybrid catfish, channel catfish and zebrafish in different *Ighμ*-coded exons found in this study: Cμ_1_ exon **(A)**, non-*Ighμ* exon **(B)**, Cμ_2_ exon **(C)**, Cμ_3_ exon **(D)**, Cμ_4_ exon **(E)** and *Ighμ* tail exon **(F)**. *, fully conserved residue of amino acids that play the same role in the same column/position.

The amino acid sequences of *Ighμ* transcripts among bighead catfish, North African catfish and their hybrid catfish were aligned and compared to published complete IgH gene loci of channel catfish (*Ictalurus punctatus*) and zebrafish (*Danio rerio*). The aligned sequences are illustrated in **Figure 2**. The results showed several important features based on the overall relative size of the amino acid sequences of general *Ighμ* transcripts, with 436 and 430 residues for the constant (Cμ_1_-Cμ_4_) domain and 44 and 41 residues for the transmembrane domains of bighead catfish and North African catfish, respectively. The mIgM and sIgM forms of the hybrid catfish were similar in size to those of the parents, bighead catfish and North African catfish (**Figure 2**). The sequence similarity of the mIgM and sIgM forms of the hybrid catfish were absolutely conserved and similar to those of the parents. These results exhibited highly important significant differences between their phenotypic characterization and relationship to their parent.

The translated amino acid sequences of the individual Cμ_1_, Cμ_2_, Cμ_3_, Cμ_4_ and transmembrane domains of bighead catfish, North African catfish and their hybrid catfish were used to define significant similarity among their relationships and evolutionary lineage. The amino acid sequences of the Cμ_1_ and Cμ_2_ domains of all catfish were similar in size, with 103 and 103 residues, respectively (**Figure 2A**). However, the Cμ_1_ and Cμ_2_ domains were slightly larger and smaller than the channel catfish Cμ_1_ and Cμ_2_ domains (102 and 104 residues, respectively) (**Figure 2A**). Additionally, bighead catfish shared amino acid similarity of Cμ_1_ and Cμ_2_ to African catfish, the hybrid catfish (mIgM), the hybrid catfish (sIgM), channel catfish and zebrafish with 82.4, 100.0, 82.4, 72.5 and 38.6% similarity for the Cμ_1_ domain and 77.2, 100.0, 727.2, 57.8 and 36.8% similarity for the Cμ_2_ domain, respectively (**Supplementary Table 1**). The amino acid similarity of the Cμ_3_ domains of bighead catfish shared 69.7, 100.0, 69.7, 45.9 and 32.9% and 74.4, 100.0, 74.4, 54.03 and 43.2% similarity in the Cμ_4_ domain to North African catfish, the hybrid catfish (mIgM), the hybrid catfish (sIgM), channel catfish and zebrafish, respectively. In addition, the amino acid similarity values of transmembrane domains of the *Ighμ* carboxyl-terminal region of bighead catfish were 97.5, 100.0, 84.0 and 76.0% for North African catfish, the hybrid catfish (mIgM), channel catfish and zebrafish, respectively. Overall, based on amino acid conservation analyses of five complete domains of *Ighμ* of bighead catfish, the Cμ_1_, Cμ_2_, Cμ_3_, Cμ_4_ and TM domains shared 75.8, 100.0, 75.8, 57.5 and 38.4% similarity with North African catfish, the hybrid catfish (mIgM), the hybrid catfish (sIgM), channel catfish and zebrafish, respectively (**Supplementary Table 1**).

**Figure 3:**
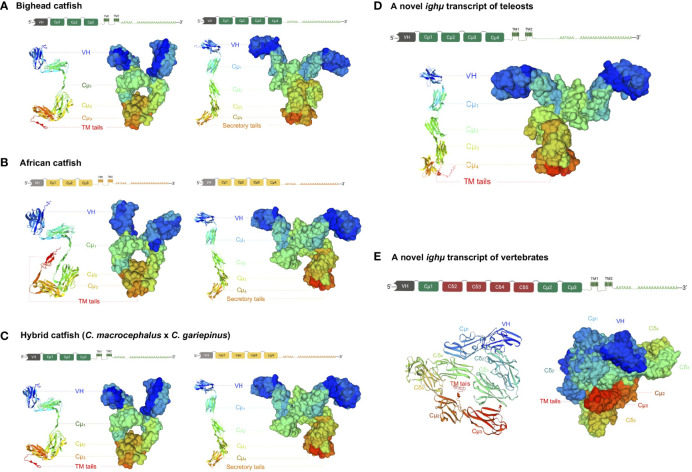
The predicted crystal protein structures of *Ighμ* transcripts expressed in the membrane and secreted forms in different catfish: bighead catfish **(A)**, North African catfish **(B)**, and their hybrid catfish **(C)**. The more complex molecules of two novel *Ighμ* transcripts found in bighead catfish are illustrated in **(D, E)**.

The conservation of the *Ighμ* domain is evident in the catfish Cμ_1_, Cμ_2_, Cμ_3_, and Cμ_4_ of *Ighμ* structures. The cysteines that likely form the intradomain disulfide bridge (*S-S*) as well as the tryptophan located within the Cμ_1_, Cμ_2_, Cμ_3_, and Cμ_4_ regions were absolutely conserved among catfish, channel catfish and zebrafish (**Figures 2A–E**). The cysteine that likely participates in the disulfide linkage of the catfish chain to the light chain was also conserved in the Cμ_1_ regions (**Figure 2A**). Additionally, the predicted results of these protein tertiary structures of *Ighμ* transcripts in bighead catfish, North African catfish and their hybrid catfish are illustrated in **Figure 3**. The *Ighμ* molecules encoded by general coding exons found in teleost and catfish mIg (Cμ_1_-Cμ_2_-Cμ_3_-TM1-TM2) and sIg (Cμ_1_-Cμ_2_-Cμ_3_-Cμ_4_) exhibited similar predicted structures in all the studied catfish (**Figures 3A–C**). Additionally, the predicted results of these protein tertiary structures of *Ighμ* transcripts of unusual splicing patterns generated novel membrane forms of *Ighμ* transcripts in teleosts [Cμ_1_-Cμ_2_-Cμ_3_-Cμ_4_-TM1-TM2] and vertebrates [Cμ_1_-(Cδ_2_-Cδ_3_-Cδ_4_-Cδ_5_)-Cμ_2_-Cμ_3_-TM1-TM2] that were larger in size and rather complex compared to other general predicted structures (**Figures 3D, E**).

**Figure 4:**
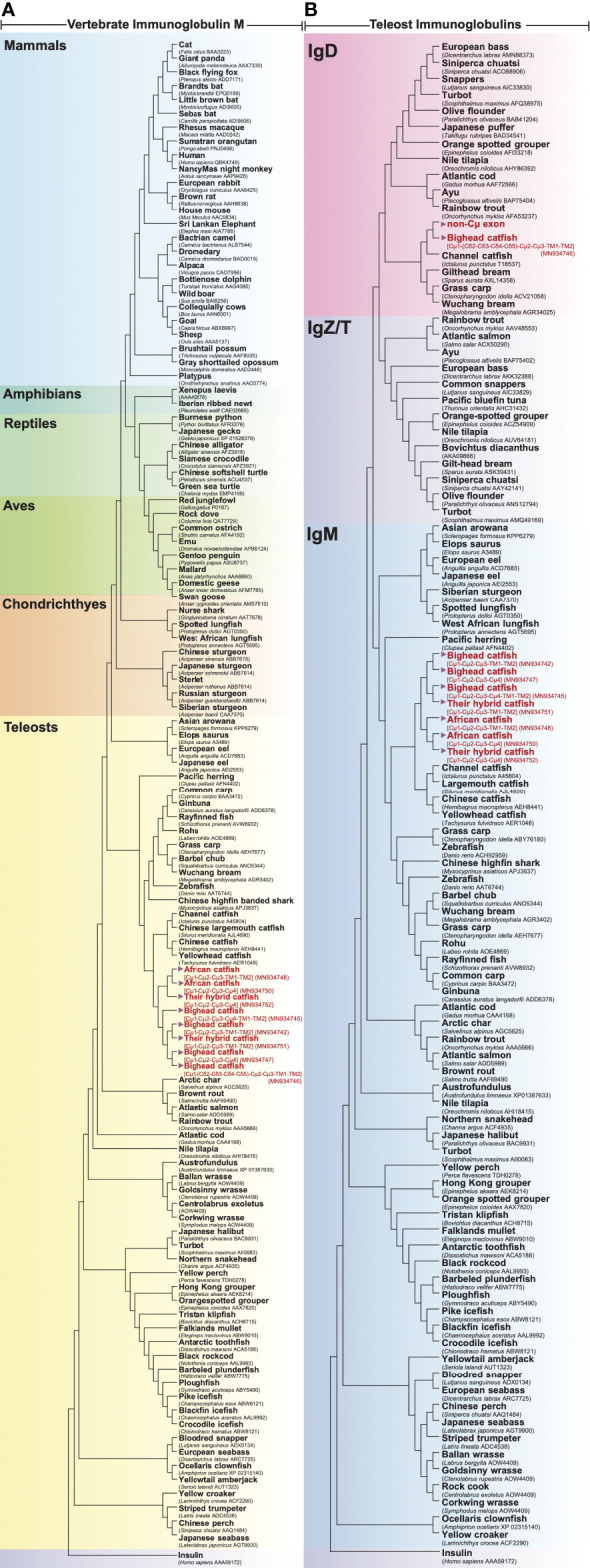
Evolutionary phylogenetic relationship of *Ighμ* between bighead catfish (NCBI accession nos. MN934742, MN934743, MN934744, MN934745, MN934746 and MN934747), North African catfish (NCBI accession nos. MN934748, MN934749 and MN934750), their hybrid catfish (NCBI accession nos. MN934751 and MN934752) and vertebrates, including mammals, amphibians, reptiles, aves, chondrichthyes and other teleosts **(A)**. Phylogenetic relationship between immunoglobulin heavy chain, IgM, IgD and IgZ/T, expressed in reported teleost fish and bighead catfish, North African catfish and their hybrid catfish **(B)**. Phylogenetic trees were constructed using neighbor-joining (NJ) algorithms with a bootstrap of 1000 replications.

The evolutionary relationships of *Ighμ* constant regions among various vertebrate species are illustrated in **Figure 4A**, and the relationships of teleost *Igh* constant regions are illustrated in **Figure 4B**. In this study, the *Clarias* catfish *Ighμ* constant domains were cladded into the catfish group of vertebrate lineages and classified into the *Ighμ* group among the reported *Igh* in fish.

### Unusual Splicing Patterns Generate Novel Membrane Forms of *Ighμ* Transcripts in Teleosts and Vertebrates

The two novel and unusual splicing patterns of the *Ighμ* membrane form were found in only bighead catfish: one was associated with a new splicing pattern among teleosts, and the other among vertebrates. The constant region of the new *Ighμ* membrane form in teleosts is encoded by four Cμ_4_ exons and directly spliced into the first transmembrane exons (Cμ_1_-Cμ_2_-Cμ_3_-Cμ_4_-TM1-TM2), as reported in other vertebrates, such as tetrapods, birds, sharks, and mammals. However, the castratory event was observed by the lack of a cryptic donor splicing site at the 3′ region of the Cμ_4_ exon, which is important for splicing to the transmembrane domain to generate the membrane form as reported previously. The predicted crystal protein structure of this molecule is illustrated in **Figure 3D**. The predicted results of the protein tertiary structures are close to the actual crystal structures of reported *Ighμ* transcripts in bighead catfish (**Figure 3A**), North African catfish (**Figure 3B**) and their hybrid catfish (**Figure 3C**).

The other novel and unusual splicing pattern of the *Ighμ* transcript was notable for vertebrates. The largest molecule of the *Ighμ* membrane-bound form was defined together with the new *Ighμ* splicing pattern among vertebrate animals. The molecular size of the molecule was approximately 92.64 kDa by encoding two major constant *IgH* loci in teleosts: Cμ and non-Cμ gene loci. The molecule encoded three Cμ_s_ and non-Cμ constant exons. The first exon of the 5′N-terminal domain was the Cμ_1_ exon of the *Ighμ* gene, which was directly spliced to the non-Cμ exons (487 residues) (**Figure 2B**) and then spliced directly to link the Cμ_2_, Cμ_3_, TM1 and TM2 exons of the *Ighμ* gene of bighead catfish. The translated amino acid sequences of the Cμ exons, including Cμ_1_, Cμ_2_, Cμ_3_, TM1 and TM2, were identical (100%) to the previously described *Ighμ* gene of bighead catfish (**Figures 1B**, **2**). In addition, the encoded non-Cμ constant region exons were studied *via* BLAST and compared to the published complete *IgH* gene loci of channel catfish and zebrafish. The translated amino acid sequences of the entire non-Cμ exons were 57.73 and 34.63% identical to the *Ighδ* gene locus of channel catfish and zebrafish, respectively (**Supplementary Table 1**). The evolutionary relationship analysis of non-Cμ exons of bighead catfish was confirmed; it was grouped into *Ighδ* among other teleosts and shared a branch with the *Ighδ* of the closely related teleost species channel catfish (**Figure 4B**). In addition, the nearest neighbors’ relationships of presumptive *Ighδ* constant exons were gilthead bream (*Sparus aurata*, AXL14358), grass carp (*Ctenopharyngodon idella*, ACV21058) and Wuchang bream (*Megalobrama amblycephala*, AGR34025). However, the presumptive non-Cμ exon sequences clustered within teleost *Ighδ* rather than *Ighμ.* The predicted protein tertiary structure of this molecule was larger in size and rather complex compared to other predicted structures (**Figure 3E**).

### Genomic Organization of the *Ighμ* Gene Locus

To gain insight into the *Ighμ* locus of *Clarias* catfish and hybrid catfish, we also defined the complete genomic organization of the gene locus. The corresponding genomic sequences encoding the *Ighμ* constant domain were amplified, cloned, sequenced and compared to the published complete *Igh* gene locus in channel catfish. Consistent with this finding, only a single copy of the genomic *Ighμ* gene locus was found in bighead catfish and North African catfish (**Figures 1E, F, H, I**, and **Supplementary Figures 10**, **11**). The complete constant region of the *Ighμ* locus, which encodes an Ig *μ* domain, contained approximately 6,793 kb (six exons, five introns and six polyadenylation signals) and 6,660 kb (six exons, five introns and six polyadenylation signals) of bighead catfish and North African catfish, respectively. However, the individual *Ighμ* loci of each bighead catfish and North African catfish were also found in the hybrid catfish, which had the transmembrane and secreted *Ighμ* transcripts, as mentioned before. The nucleotide identity between the two *Ighμ* gene loci in the hybrid catfish was identical to those of the parents, bighead catfish and African catfish (**Figures 1G, J** and **Supplementary Figures 10**, **11**).

### Diversity Analysis of the Variable Domain (VH) of *Ighμ* Genes

**Figure 5:**
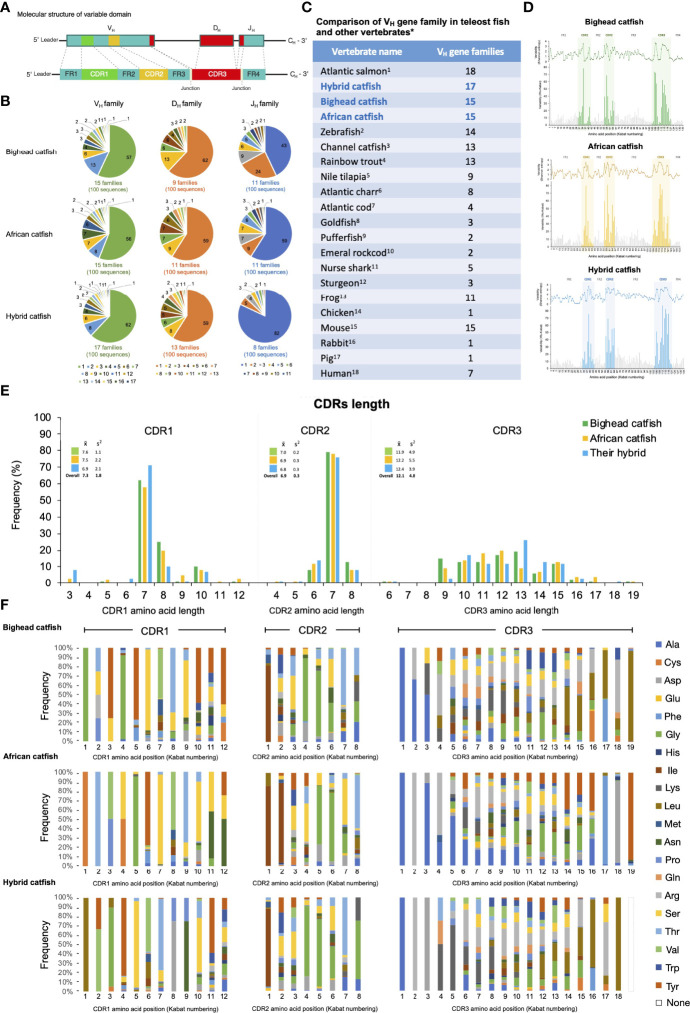
Representation of comparative diversity analyses of the VH domains of *Ighμ* bighead catfish, North African catfish and their hybrid catfish. General structures of the VH domain correspond to the V_H_, D_H_, and J_H_ segments that consist of the FR1, CDR1, FR2, CDR2, FR3, CDR3 and FR4 regions found in all vertebrates **(A)**. Comparative classification of the V_H_, D_H_, and J_H_ gene segments in bighead catfish, North African catfish and their hybrid catfish **(B)**. Comparison of V_H_ gene family members among reported vertebrates **(C)**. Degree of sequence variability of the VH repertoire in bighead catfish, North African catfish and their hybrid catfish, according to the Shannon (1948) and Kabat and Wu (1971) methods **(D)**. Comparison of amino acid length in CDR1, CDR2, and CDR3 of bighead catfish, North African catfish and their hybrid catfish **(E)**, x̅: average nucleotide length, S^2^: variance distribution. Percent frequency of amino acid variability and composition of CDR1 CDR2 and CDR3 regions of bighead catfish, North African catfish and their hybrid catfish **(F)**. The NCBI accession numbers of VH nucleotide sequences are MN934442- MN934541, MN934542- MN934641 and MN934642- MN934741 for bighead catfish, North African catfish and their hybrid catfish, respectively.^1^Yasuike et al., 2010 (44); ^2^Danilova et al., 2005 (13); ^3^Yang et al., 2003 (45); ^4^Brown et al., 2006 (46); ^5^Phuyindee et al., 2015 (47); ^6^Andersson and Matsunaga, 1998 (48); ^7^Stenvik et al., 2000 (49); ^8^Wilson et al., 1991 (31); ^9^Peixoto and Brenner, 2000 (50); ^10^Coscia and Oreste, 2003 (22); ^11^Rumfelt et al., 2004 (51); ^12^Lundqvist et al., 1998 (52); ^13^Haire et al., 1990 (53);^14^Ota and Nei, 1995 (54); ^15^Mainville et al., 1996 (55); ^16^Mage et al., 1984 (56); ^17^Sun et al., 1994 (57) and ^18^Matsuda et al., 1998 (58).

A total of 100 unique nonredundant clones of each catfish were characterized for VH sequences of the *Ighμ* gene. The functional regions of VH encoded by the V_H_, D_H_ and J_H_ segments were identified, and analysis of framework regions (FWs) and complementarity-determining regions (CDRs) was also performed (**Figure 5A**) according to the international immunogenetics (IMGT) information numbering system for Ig structural definition (59).

Based on 80% nucleotide identity, the functional V_H_ segments were classified into *15*, *15* and *17* families for bighead catfish, North African catfish and their hybrid catfish, respectively. Between the most 3′ V_H_ segment and the 5′ J_H_ segment, 9, 11 and 13 functional D_H_ segment families were defined in bighead catfish, North African catfish and their hybrid catfish, respectively. Altogether, the functional J_H_ segments of catfish VH have been identified. There were 11, 11 and 8 families of bighead catfish, North African catfish and their hybrid catfish, respectively (**Figure 5B**). The comparisons of the V_H_ gene family among catfish in this study and among reported vertebrates are illustrated in **Figures 5B, C**, respectively. The NCBI accession numbers of VH nucleotide sequences are MN934442-MN934541, MN934542-MN934641 and MN934642-MN934741 for bighead catfish, North African catfish and their hybrid catfish, respectively. In addition, adding P- and N-nucleotides was predominantly observed at the junction of the V and J segments for the generation of V(D)J junctional diversity in bighead catfish, North African catfish and their hybrid catfish (**Supplementary Figures 12**–**14**).

### Analysis of Framework Regions (FWs) and Complementarity-Determining Regions (CDRs)

To determine the extent of diversification in the VH repertoire of the *Ighμ* gene, we calculated the length variation and percent deviations of amino acids in each position of the FWs and CDRs and compared them to those of germline VH genes from the international ImMunoGeneTics information system^®^ (IMGT^®^, http://www.imgt.org) (60).

The FWs of bighead catfish, North African catfish and their hybrid catfish were similar in length and diversity of amino acids. The lengths of amino acids of FW1, FW2, FW3 and FW4 in all catfish were approximately 33, 14, 36 and 14 residues, respectively (**Figure 5D**). To examine the degree of sequence variability of the VH repertoire in *Clarias* catfish, plots of the variability according to the Shannon (1948) (40) and Kabat and Wu (1971) (41) methods were generated. The overall amino acid sequence variability of the VH region was mostly confined to the CDRs and particularly the CDR3s. In addition, the amino acid variability among CDRs (CDR1, CDR2 and CDR3) was highly similar to that of FR1 to FR4 of all studied catfish. Moreover, the comparisons of variability plots of VH sequences among the studied *Clarias* catfish were closely related in sequence patterns and variability level to each other based on both the Shannon and Kabat and Wu methods (**Figure 5D**). Shannon entropy plots of all catfish revealed increased diversity at amino acid positions 31 to 42 for CDR1, 58 to 65 for CDR2 and 102 to 120 for CDR3 (**Figure 5D**). They were generally related to the traditional definitions using the Kabat numbering convention (**Figure 5D**).

The amino acid length diagrams of CDRs of bighead catfish, North African catfish and their hybrid catfish in **Figure 5E** indicate that the amino acid length of CDR1 ranged from 3 to 12 residues with an average of 7.6, 7.5 and 6.9 residues, respectively. The most common length was seven for all catfish, with approximately 58 to 71% frequency.

Within nonredundant CDR1 (n = 100) sequences of each *Clarias* catfish, 6 major amino acids were observed in bighead catfish, which accounted for 79.7% of all residues: Ala (7.7%), Gly (16.5%), Ser (18.9%), Thr (9.1%), Val (4.1%) and Tyr (23.1%). For North African catfish, 8 major amino acids were observed, which accounted for 93.7% of all residues: Cys (11.7%), Gly (8.5%), Asn (8.0%), Pro (4.0%), Ser (30.2%), Thr (14.5%), Val (8.7%), and Tyr (7.9%). For their hybrid catfish, 7 major amino acids were observed, which accounted for 75.2% of all residues: Asp (6.8%), Gly (8.2%), Leu (10.2%), Ser (17.3%), Thr (8.1%), Val (9.7%), and Tyr (14.6%) (**Figure 5F**).

The amino acid sequences of the CDR2 were shorter overall than those of CDR1, which were 4 to 8 residues in bighead catfish, North African catfish and their hybrid catfish with average amino acid lengths of 7.0, 6.9 and 6.8 residues, respectively. Approximately 76 to 79% of nonredundant CDR2s were 7 amino acid residues in length. Shannon entropy plots of amino acid variation of nonredundant CDR1 and CDR2 were mostly similar in diversity to those of the related V_H_ families within the species and among *Clarias* species. Within 8 residues in length of CDR2 revealed highly conserved residues at position one of the CDR1s (**Figure 5F**).

Six major amino acids were observed in nonredundant CDR2s (n = 100) sequences of bighead catfish, which accounted for 78.4% of all residues: Ala (5.4%), Asp (5.8%), Gly (25.3%), Ile (12.4%), Ser (11.7%) and Thr (17.5%). For North African catfish, 5 major amino acids were observed, which accounted for 78.2% of all residues: Asp (5.6%), Gly (21.0%), Ile (22.9%), Ser (17.4%) and Thr (11.1%). For their hybrid catfish, 5 major amino acids were also observed, which accounted for 73.2% of all residues: Asp (5.3%), Gly (31.8%), Ile (12.1%), Ser (11.3%) and Thr (12.4%) (**Figure 5F**).

The length distributions of the *Clarias* catfish CDR3 repertoire varied from 6 to 19 residues with averages of 11.9, 12.2 and 12.4 residues for bighead catfish, North African catfish and their hybrid catfish, respectively. The most common length was 10 to 15 residues for all catfish, with approximately 13 to 26% frequency. Analysis of amino acid frequencies for nonredundant CDR3s across all individual catfish revealed that the highest mutation rates in the VH repertoire were observed within CDR3 for all studied catfish. Highly conserved amino acid residues at positions one and two were observed in the CDR3 of all catfish. Seven major amino acids were observed in bighead catfish, which accounted for 66.3% of all residues: Ala (13.4%), Lys (7.1%), Leu (17.7%), Pro (3.6%), Gln (3.1%), Arg (17.1%) and Trp (4.0%). For North African catfish, 7 major amino acids were identified, which accounted for 80.3% of all residues: Ala (22.6%), Asp (7.5%), Gly (5.8%), His (9.5%), Ser (16.0%) Thr (4.3%) and Yyr (14.4%). For their hybrid catfish, 8 major amino acids were observed, which accounted for 77.4% of all residues: Ala (7.9%), Asp (5.9%), Gly (5.5%), Lys (10.7%), Leu (16.8%), Arg (21.0%), Ser (5.0%) and Thr (4.3%). Notably, three amino acids, Gly, Ser and Thr, were predominantly distributed in all CDR1s, CDR2s and CDR3s of all catfish in this study (**Figure 5F**).

### Expression and Tissue Distribution of *Ighμ* Genes

**Figure 6:**
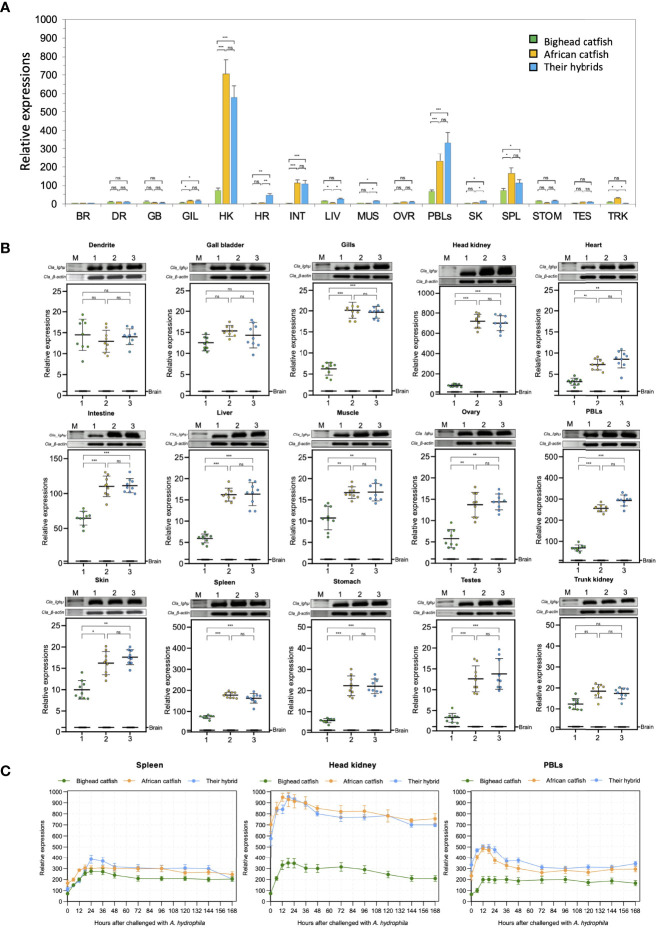
Representation of the overall relative expression and distribution of *Ighμ* in bighead catfish, North African catfish and their hybrid catfish in sixteen tissues **(A)**. Comparison of the relative expression of *Ighμ* in sixteen different tissues **(B)**. Expression responses of *Ighμ* in immune-related organs, including the spleen, head kidney and PBLs, after intraperitoneal injection of a virulent pathogen, *Aeromonas hydrophila*, of bighead catfish, North African catfish and their hybrid catfish **(C)**. The relative expression levels of *Ighμ* genes were calculated using *β-actin* as a reference gene. All quantitative data are presented as the mean ± standard deviation (SD). The levels of statistical significance are indicated by *(*P*<0.05), **(*P*<0.01) or ***(*P*<0.001). The brain RT was used as the calibrator. All experiments are done in triplicate (n = 3). ns, not significant.

To examine the distribution of *Ighμ* in *Clarias* catfish, we examined the potential functional coding segment of *Ighμ*, the Cμ_2_ region, in sixteen tissues of catfish. *Ighμ* was highly expressed in the head kidney, spleen and peripheral blood lymphocytes (PBLs) in all studied catfish (**Figure 6A**). The relative expression levels reached approximately 60-, 700- and 600-fold the accustomed expression in head kidney, an important fish immune response organ, of bighead catfish, North African catfish and their hybrid catfish, respectively. However, the lowest expression was also observed in various tissues, including the brain, dendrite, gallbladder, gills, heart, liver, muscle, ovary, skin, stomach, testes and trunk kidney, in all studied catfish (**Figures 6A, B**). Highly significant expression levels were observed in North African catfish and the hybrid catfish in several tissues, including the gills, head kidney, heart, intestine, liver, muscle, ovary, PBLs, skin, spleen, stomach, and testes, compared to those in bighead catfish (**Figure 6B**). No significant difference was observed in tissues of North African catfish and the hybrid catfish. However, the relative expression of *Ighμ* was not significantly different in dendrites, gall bladder or trunk kidney among all studied catfish (**Figure 6B**).

Intraperitoneal injection of virulent *Aeromonas hydrophila* clearly exhibited the pattern of immune responses of catfish, and its significance was defined among the three catfish. In the spleen, the expression of *Ighμ* was moderately increased approximately 200-400-fold after 12–168 hours (hr) of pathogen exposure for all catfish. For the head kidney, the expression of *Ighμ* was strongly increased approximately 200–300-, 850–950-, and 800–950-fold after 12–24 hr. of pathogen exposure for bighead catfish, North African catfish and their hybrid catfish, respectively. In the PBLs, *Ighμ* expression showed significant upregulation of approximately 200-, 450- and 500-fold after 12–24 hr. of exposure for bighead catfish, North African catfish and their hybrid catfish, respectively. In addition, an approximate upregulation of 200–300-fold was also observed in all catfish after 12–24 hr. of exposure (**Figure 6C**).

**Figure 7:**
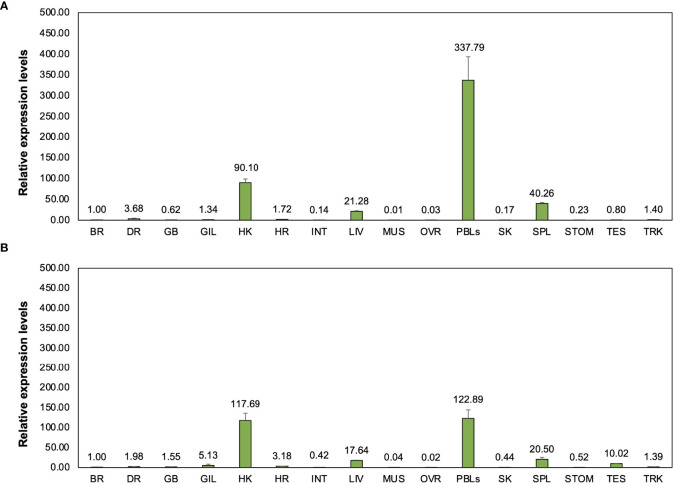
Representation of relative expression levels and distribution of two novel *Ighμ* molecules in bighead catfish including *Cm*_mIgM2 **(A)** and *Cm*_mIgM3 **(B)** in sixteen tissues. All experiments are done in triplicate (n=3).

The relative expression of two novel transcripts, including *Cm*_mIgM2 and *Cm*_mIgM3 in bighead catfish tissues showed highly expression levels compared to brain, approximately 337- and 122-, 90- and 177-, 40- and 20-, 21- and 17-fold, in PBLs, head kidney, spleen, and liver of *Cm*_mIgM2 and *Cm*_mIgM3, respectively (**Figures 7A, B**).

## Discussion

The specific features of *Ighμ* constant region transcripts found in bighead catfish, North African catfish and their hybrid catfish conform to the patterns of organization reported previously in other vertebrate and teleost *Ighμ* genes. The *Ighμ* constant regions of the secreted form of *Ighμ* are a typically spliced pattern, as found in other teleosts, which are encoded by four Cμ protein domains, Cμ_1_, Cμ_2_, Cμ_3_ and Cμ_4_, without a transmembrane protein domain at the carboxyl-terminal region (7, 61). In addition, the general mRNA splicing patterns to generate the membrane form of *Ighμ* were found in all studied catfish by direct splicing from Cμ_3_ to the first transmembrane exon with the lack of a cryptic donor splicing site (T/C/G↓GGTAAA), as reported in the 3′ region of the Cμ_4_ exon in mammalian, shark, and amphibian mIg molecules. In this study, two domains of transmembrane proteins of *Ighμ* were found in all studied catfish as well as in channel catfish and other teleosts (5, 14, 24, 29).

Two unexpected and unusual *Ighμ* constant region transcripts were found in bighead catfish. One is a novel *Ighμ* transcript for teleosts, and the other is a novel transcript for vertebrates. The form is unusual in that the transmembrane domains are directly spliced to exon Cμ_4_ to create a membrane-bound form of *Ighμ* that did not seem to conform to any patterns of the membrane form of the *Ighμ* structure previously reported in teleosts. The molecule corresponded with Cμ_1_-Cμ_2_-Cμ_3_-Cμ_4_-TM1-TM2. Although this molecular pattern feature is typical of most mammals and vertebrates examined previously (62, 63), the cryptic donor splicing site at the 3′ region of the Cμ_4_ exon, the acceptor site of the first transmembrane (TM1) exon (62, 64), was not found in the case of this study. In this regard, the presence of the Cμ_4_ exon between Cμ_3_ and the first transmembrane domain of the *Ighμ* membrane-bound form produced a unique characteristic of the mIg form as a novel membrane receptor feature of B cells in bighead catfish and teleosts.

Another important *Ighμ* transcript was discovered in bighead catfish, and it is interesting in that the membrane-bound form of *Ighμ* was encoded by two major exon domains, Cμ and non-Cμ exon domains. The molecular pattern was obviously characterized as Cμ_1_-(non-Cμ)-Cμ_2_-Cμ_3_-TM1-TM2. The presence of 487 amino acid residues of the non-Cμ exon between the Cμ_1_ and Cμ_2_ regions of the typical membrane-bound form of *Ighμ* was spliced directly to the end of the Cμ_1_ exon without any additional or lacking amino acids. Thus far, the non-Cμ exon was defined compared to the most closely and distinctly related published *Igh* locus, channel catfish (*Ictalurus punctatus*) (63, 65) and zebrafish (*Danio rerio*) (13, 66). The amino acid similarity of the non-Cμ exon was 57.73% and 34.63% to the *Ighδ* gene loci of channel catfish and zebrafish, respectively. It is also characterized as four *Ighδ* constant exons based on the *Ighδ* gene locus of channel catfish, C_δ2_-C_δ3_-C_δ4_-C_δ5_. There were 79, 220, 94 and 94 amino acid residues, respectively (63, 65). In this regard, it is interesting that the 3′ end of non-Cμ, C_δ5,_ was directly spliced to the Cμ_2_-Cμ_3_-TM1-TM2 exons with the identical polyadenylation signal at the C-terminus of the described typically membrane-bound form of the *Ighμ* molecule in bighead catfish. The evolutionary relationship analysis of this novel membrane-bound form seemed to confirm the identification by grouping into the *Ighδ* clade among other teleosts and sharing a branch to the *Ighδ* of the closely related teleost species, channel catfish (**Figure 4**). However, the presumptive non-Cμ exon sequences clustered within the teleost *Ighδ* rather than *Ighμ* gene domains. The new pattern of mRNA splicing of the *Ighμ* membrane formed by two major *Ighμ* and *Ighδ* constant exons could be defined as Cμ_1_-Cδ2-Cδ3-Cδ4-Cδ5-Cμ_2_-Cμ_3_-TM1 and TM2. The presence of encoding *Ighδ* constant exons inside the *Ighμ* molecule suggests that this was a novel splicing pattern among vertebrate animals that has never been present in any class of vertebrates thus far (5, 9, 25, 29, 66). However, the unusual splicing of the two patterns was not found in either North African catfish or the hybrid catfish. Therefore, it is possible that bighead catfish express *Ighμ* membrane receptors with at least three different mRNA splicing patterns.

Inside the gene organizations of constant *Ighμ* regions, the lengths of the five introns separating these six exons seem to be similar to those of the closely related species channel catfish^42^ and quite different from those of other vertebrates. The intron separating Cμ_1_ and Cμ_2_ was approximately 1.7 kb for bighead catfish, 1.8 kb for North African catfish and 1.7 kb for channel catfish (63). *Xenopus* also exhibited a long intron between Cμ_1_ and Cμ_2_ of approximately 1.6 kb (67, 68). Smaller introns separating Cμ_2_ to Cμ_3_ and Cμ_3_ to Cμ_4_ of approximately 0.2–0.4 kb and 0.2–0.3 kb were found in bighead catfish and North African catfish, respectively, and were also identified in channel catfish (63). However, a longer intron (approximately 1.4 kb) between exons Cμ_2_ and Cμ_3_ was found in the horn shark *Heterodontus francisci* (69). An extensive distance between Cμ_4_ and the first transmembrane domain exons of approximately 1–3 kb seems to be observed in bighead catfish, North African catfish and all reported vertebrates, such as mouse, horned shark and channel catfish (63, 67–69) . The introns separating Cμ_1_, Cμ_2_, Cμ_3_, and Cμ_4_ in mouse are all less than 0.5 kb (70, 71). In addition, a large intron separating TM1 and TM2 exons was also approximately 1.3 kb and 1.2 kb for bighead catfish and North African catfish, respectively, as found in *Xenopus* (approximately 1.1 kb) and channel catfish (approximately 2.3 kb) (63, 67, 69) . On the basis of the obtained data, this *Ighμ* gene organization was characteristically generalized to all teleost fish.

The gene localization of the *Ighμ* gene region of the IgH constant region locus of bighead catfish, North African catfish and their hybrid catfish confirms that bighead catfish and North African catfish possess only a single copy of this gene, whereas their hybrid catfish possesses two copies of this gene (**Figure 1J**). The finding that the *Clarias* catfish *Ighμ* gene is most likely found in a single copy in the genome suggests that it may be common to all teleost fish, e.g., in channel catfish (72) and several other teleosts (26, 73, 74). The obtained *Ighμ* constant gene organization of the hybrid catfish clearly showed that the sequences were identical to those of the parents. The presence of two copies of the *Ighμ* gene in the hybrid catfish may be normal in diploid hybrid animals. In this regard, it is interesting that the *Ighμ* transcripts of the hybrid catfish are processed from different gene loci, and the membrane-bound and secretory forms were made up of the *Ighμ* gene of bighead catfish and North African catfish, respectively. The degree similarity of the amino acid was also identical to those of the parents. This available evidence would seem thus far to support the disease resistance characteristics of North African catfish and the hybrid catfish in several research reports. This suggests that the soluble effector molecules secreted from *Ighμ* of North African catfish and the hybrid catfish may be strongly involved in specific immune responses, such as toxin or microbe neutralization, opsonization (immunophagocytosis), antibody-dependent cell-mediated cytotoxicity (ADCC), and complement activation (immunolysis) (1, 75).

Furthermore, the ability of Ig to interact and bind antigen was involved in the variable domains of *Ighμ* molecules. Specific pathogens or antigens are recognized *via* the fragment antigen-binding (Fab) variable region (49). Additional analysis of the VH domain provided extensive insight. In our findings, the overall structures of bighead catfish, North African catfish and their hybrid catfish VH region corresponded to the V_H,_ D_H,_ and J_H_ segments that consist of FR1, CDR1, FR2, CDR2, FR3, CDR3 and FR4, as reported in all vertebrates examined previously (49, 76).

Analysis of the V_H_, D_H_ and J_H_ family classification based on their 80% similarities of amino acids interestingly demonstrated that a large number of V_H_ gene families were defined in all studied catfish, of approximately 15–17 family members from one hundred nonredundant clones. There are similarities in some teleosts and some mammals, e.g., Atlantic salmon, zebrafish, and mice (14–18 families). However, there were also higher levels in the V_H_ gene family than in vertebrates (**Figure 5C**) (13, 44, 58). Much less is known and reported about the D_H_ and J_H_ segments of teleosts than about the V_H_ of teleosts. Bighead catfish, North African catfish and their hybrid catfish process approximately 9, 11, and 13 families and 11, 11, and 8 families for a hundred nonredundant clones of D_H_ and J_H_ segments, respectively, compared to 9 in channel catfish, 4 in mouse, 6 in human, 6 in *O. mykiss*, and 2 in *G. morhua* for the J_H_ segment (73). To date, there have been few reports of D_H_ region diversity in vertebrates and teleosts, and significant variability in D_H_ segments can be inferred from the generation of variability in CDR3 defined in the different cDNA clone sequences (49, 73). This available evidence indicates that the mechanisms of VH diversity generalization of *Ighμ* are made up of several gene family members that are necessary for generating several distinct VH repertoires in all studied catfish. In addition, junctional diversity occurs at the junction of the V_H_-D_H_ and D_H_-J_H_ boundaries. These regions code for CDR3. Diversity is increased by the addition of P-nucleotides and N-nucleotides (49). P-nucleotides are often made-up palindromic sequences and added to the ends of the asymmetrical DNA strand. Moreover, the random nontemplate-encoded addition of 2 to 20 base pairs or N nucleotides changes the amino acid sequence in the hypervariable CDR3. P- and N-nucleotides are principally found in the V_H_-D_H_ and D_H_-J_H_ junctions of the assembled heavy chain gene (49, 76). Furthermore, it was suggested that inversion (D-D joining), nucleotide deletion, heavy-light chain pairing and somatic hypermutation contribute to diversity in VH antigenic recognition after naive B cells are released to the periphery and encounter antigens (49, 73). Based on the findings in the present study, these mechanisms may be used to generate diverse VH repertoires in bighead catfish, North African catfish, and their hybrid catfish.

Analysis of the CDR3s provided strong evidence that the CDR3 of all studied catfish is longer than those of Atlantic cod, trout, frog and mouse but shorter than those of rabbit, human and cattle (13, 44, 49, 58). In addition, overall analysis of the VH domain of *Ighμ* indicated that the amino acid deviation in the CDRs was higher than that in the FWs in all catfish. Notably, these findings generally indicated predominant conservation in the VH repertoire (49, 73). However, the calculated amino acid variability and composition of all CDRs in VH interestingly demonstrated that high variability plots and variability in amino acid composition were observed in all CDR1s, CDR2s and CDR3s of all studied catfish compared to those of reported vertebrates (49, 77, 78). Based on our findings, the potential for increasing the structural variability of the VH domain in *Clarias* catfish and their hybrids seems to be higher than that of teleosts, particularly given the number of V_H_ and J_H_ family members, long length of CDR3, and high variability in all CDR1s, CDR2s and CDR3s. This may be a general feature of *Clarias* catfish, which has never been reported thus far.

The functional expression of *Ighμ* in bighead catfish, North African catfish and their hybrid catfish was examined in different tissues. The *Ighμ* gene of all studied catfish and two novel *Ighμ* molecules of bighead catfish were typically expressed in any organ and predominantly expressed in immune-related organs, especially the head kidney, PBLs, spleen and liver tissues, which is in accordance with previous studies (13, 79). High expression of the two novel transcripts in immune-involved organs may indicate specific unknown functions that need further investigation.

In our study, significant responses of *Ighμ* expression among *Clarias* catfish and the hybrid catfish were observed after bacterial exposure. North African catfish and the hybrid catfish exhibited approximately 900–950-fold relative expression levels in head kidney and 400–500-fold in PBLs; these levels are much higher than those of bighead catfish by at least 2–2.5-fold changes. The head kidney in teleosts is considered a primary lymphoid organ similar to the mammalian bone marrow, a major hemopoietic organ and site of Ig and other immune cell production in teleosts (15). *A. hydrophila* is a significant pathogen of *Clarias* catfish in Thailand. It is highly pathogenic due to the presence of hemolysin and aerolysin virulence genes, which demonstrate high virulence in *Clarias* catfish acute septicemia. A virulent strain could cause death within 24 hrs. and resulted in abdominal effusion and varying degrees of internal organ hemorrhage. The precise mechanism by which specific IgM immune responses rapidly increased after 12-24 hrs. post-exposure is still uncertain in the *A. hydrophila* challenge experiment. It could be suggested in two ways. First, as a characteristic of acute septicemia, the virulent bacteria evenly divide speedily in the blood and are rapidly circulated to the major immune-related tissues such as the head and kidney, activating the chemokine signaling pathway and triggering an inflammatory response, as well as upregulating IgM in the host. Second, prior to conducting the experiment, all studied catfish were exposed to *A. hydrophila* in their surrounding environment, resulting in a strongly rapid upregulation of IgM specific to *A. hydrophila* 12-24 hrs. post-exposure.

Unexpectedly, the pattern of *Ighμ* expression in hybrid catfish is consistent with their fatherhood of North African catfish. This evidence may imply a long mysterious question regarding the well-known characteristics of the bacterial resistance of North African catfish and the hybrid catfish, which normally are much higher than those of bighead catfish.

These results indicate that the *Ighμ* transcripts in bighead catfish, North African catfish and the hybrid catfish are typically produced by splicing patterns of mRNA reported in all teleosts. Notably, in bighead catfish, two more unusual spicing *Ighμ* transcripts of membrane-bound forms, 1) Cμ_1_-Cμ_2_-Cμ_3_-Cμ_4-_TM1-TM2 and 2) Cμ_1_-(Cδ_2_-Cδ_3_-Cδ_4_-Cδ_5_)-Cμ_2_-Cμ_3_-TM1-TM2, represent novel patterns of mRNA splicing of the *Ighμ* membrane form for teleosts and vertebrates, respectively. However, the reasons for the addition of *Ighδ* to *Ighμ* molecules are still unknown. All *Ighμ* transcripts were produced from a single gene copy in individual bighead catfish and North African catfish. In addition, the different *Ighμ* transcripts, membrane and secreted forms, of diploid hybrid catfish consisted of two copies of the *Ighμ* gene from the parents, bighead catfish and North African catfish. Extensive insight into the VH domain could generate a number of distinct structural variabilities in the VH domain in *Clarias* catfish and the hybrid catfish. The overall functional significance of *Ighμ* in hybrid catfish is mostly similar to that in North African catfish.

## Data Availability Statement

The datasets presented in this study can be found in online repositories. The names of the repository/repositories and accession number(s) can be found in the article/**Supplementary Material**.

## Ethics Statement

The animal study was reviewed and approved by The Animal Ethics Committee, Kasetsart University, Thailand (Ethics ID: ACKU61-FIS-004). Written informed consent was obtained from the owners for the participation of their animals in this study.

## Author Contributions

AB designed and performed all the experiments and wrote the original manuscript. UN-N and PS revised the manuscript. PS designed the experiments and revised the manuscript. All authors listed have read and approved the manuscript for publication.

## Funding

This work was financially supported by the Thailand Research Fund (TRF) and the Betagro Science Center, Thailand. This study is part of the project entitled “Genetics and Biotechnology for Improvement of Aquatic Animal Production” (contract number DPG5980003) awarded to Professor Uthairat Na-Nakorn under the “Distinguished Research Professor 2016 Award.”

## Conflict of Interest

The authors declare that this research was conducted in the absence of any commercial or financial relationship that could be construed as a potential conflict of interest.

## Publisher’s Note

All claims expressed in this article are solely those of the authors and do not necessarily represent those of their affiliated organizations, or those of the publisher, the editors and the reviewers. Any product that may be evaluated in this article, or claim that may be made by its manufacturer, is not guaranteed or endorsed by the publisher.

## References

[1] Burton DR Woof JM. Human Antibody Effector Function. Adv Immunol (1992) 51:1–84. doi: 10.1016/S0065-2776(08)60486-1 1502974

[2] Warr GW. The Immunoglobulin Genes of Fish. Dev Comp Immunol (1995) 19:1–12. doi: 10.1016/0145-305X(94)00052-H 7615133

[3] Coscia MR Varriale S De Santi C Giacomelli S Oreste U. Evolution of the Antarctic Teleost Immunoglobulin Heavy Chain Gene. Mol Phylogenet Evol (2010) 55:226–33. doi: 10.1016/j.ympev.2009.09.033 19800977

[4] Stavnezer J Amemiya CT. Evolution of Isotype Switching. Semin Immunol (2004) 16:257–75. doi: 10.1016/j.smim.2004.08.005 15522624

[5] Hikima J Jung TS Aoki T. Immunoglobulin Genes and Their Transcriptional Control in Teleosts. Dev Comp Immunol (2011) 35:924–36. doi: 10.1016/j.dci.2010.10.011 21078341

[6] Ohno S. Immunoglobulins Vol. 197-204. Litman GW Good RA, editors. New York, NY, USA: Springer USA (1978).

[7] Wilson MR van Ravenstein E Miller NW Clem LW Middleton DL Warr GW. cDNA Sequences and Organization of IgM Heavy Chain Genes in Two Holostean Fish. Dev Comp Immunol (1995) 19:153–64. doi: 10.1016/0145-305X(94)00063-L 7556802

[8] Roux KH Greenberg AS Greene L Strelets L Avila D McKinney EC. Structural Analysis of the Nurse Shark (New) Antigen Receptor (NAR): Molecular Convergence of NAR and Unusual Mammalian Immunoglobulins. Proc Natl Acad Sci USA (1998) 95:11804–9. doi: 10.1073/pnas.95.20.11804 PMC21721 9751746

[9] Schaerlinger B Bascove M Frippiat J-P. A New Isotype of Immunoglobulin Heavy Chain in the Urodele Amphibian *Pleurodeles Waltl* Predominantly Expressed in Larvae. Mol Immunol (2008) 45:776–86. doi: 10.1016/j.molimm.2007.06.356 17681605

[10] Golub R Charlemagne J. Structure, Diversity, and Repertoire of VH Families in the Mexican Axolotl. J Immunol (1998) 160:1233–9. 9570539

[11] Hsu E Criscitiello MF. Diverse Immunoglobulin Light Chain Organizations in Fish Retain Potential to Revise B Cell Receptor Specificities. J Immunol (2006) 177:2452–62. doi: 10.4049/jimmunol.177.4.2452 PMC3129705 16888007

[12] Litman GW Anderson MK Rast JP. Evolution of Antigen Binding Receptors. Annu Rev Immunol (1999) 17:109–47. doi: 10.1146/annurev.immunol.17.1.109 10358755

[13] Danilova N Bussmann J Jekosch K Steiner LA. The Immunoglobulin Heavy-Chain Locus in Zebrafish: Identification and Expression of a Previously Unknown Isotype, Immunoglobulin Z. Nat Immunol (2005) 6:295–302. doi: 10.1038/ni1166 15685175

[14] Savan R Aman A Nakao M Watanuki H Sakai M. Discovery of a Novel Immunoglobulin Heavy Chain Gene Chimera From Common Carp (*Cyprinus Carpio* L.). Immunogenetics (2005) 57:458–63. doi: 10.1007/s00251-005-0015-z 16025325

[15] Bengtén E Quiniou S Hikima J Waldbieser G Warr GW Miller NW. Structure of the Catfish IGH Locus: Analysis of the Region Including the Single Functional IGHM Gene. Immunogenetics (2006) 58:831–44. doi: 10.1007/s00251-006-0139-9 16941126

[16] Saha NR Suetake H Suzuki Y. Analysis and Characterization of the Expression of the Secretory and Membrane Forms of IgM Heavy Chains in the Pufferfish. Takifugu rubripes. Mol Immunol (2005) 42:113–24. doi: 10.1016/j.molimm.2004.06.034 15488950

[17] Oreste U Coscia M. Specific Features of Immunoglobulin VH Genes of the Antarctic Teleost. Trematomus bernacchii. Gene (2002) 295:199–204. doi: 10.1016/S0378-1119(02)00686-8 12354654

[18] Merlino A Varriale S Coscia MR Mazzarella L Oreste U. Structure and Dimerization of the Teleost Transmembrane Immunoglobulin Region. J Mol Graph Model (2008) 27:401–7. doi: 10.1016/j.jmgm.2008.07.001 18760646

[19] Magor BG Wilson MR Miller NW Clem LW Middleton DL Warr GW. An Ig Heavy Chain Enhancer of the Channel Catfish *Ictalurus Punctatus*: Evolutionary Conservation of Function But Not Structure. J Immunol (1994) 153:5556–63. 7989757

[20] Hordvik I Voie AM Glette J Male R Endresen C. Cloning and Sequence Analysis of Two Isotypic IgM Heavy Chain Genes From Atlantic Salmon, *Salmo Salar* L. Eur J Immunol (1992) 22:2957–62. doi: 10.1002/eji.1830221130 1425919

[21] Ghaffari SH Lobb CJ. Cloning and Sequence Analysis of Channel Catfish Heavy Chain cDNA Indicate Phylogenetic Diversity Within the IgM Immunoglobulin Family. J Immunol (1989) 142:1356–65. 2492581

[22] Coscia MR Oreste U. Limited Diversity of the Immunoglobulin Heavy Chain Variable Domain of the Emerald Rock Cod *Trematomus Bernacchii* . Fish Shellfish Immunol (2003) 14:71–92. doi: 10.1006/fsim.2002.0418 12547627

[23] Cheng CA John JA Wu MS Lee CY Lin CH Lin CH. Characterization of Serum Immunoglobulin M of Grouper and cDNA Cloning of Its Heavy Chain. Vet Immunol Immunopathol (2006) 109:255–65. doi: 10.1016/j.vetimm.2005.08.029 16199094

[24] Bengten E Leanderson T Pilstrom L. Immunoglobulin Heavy Chain cDNA From the Teleost Atlantic Cod (*Gadus Morhua* L.): Nucleotide Sequences of Secretory and Membrane Form Show an Unusual Splicing Pattern. Eur J Immunol (1991) 21:3027–33. doi: 10.1002/eji.1830211219 1748150

[25] Andersson E Peixoto B Tormanen V Matsunaga T. Evolution of the Immunoglobulin M Constant Region Genes of Salmonid Fish, Rainbow Trout (*Oncorhynchus Mykiss*) and Arctic Charr (*Salvelinus Alpinus*): Implications Concerning Divergence Time of Species. Immunogenetics (1995) 41:312–5. doi: 10.1007/BF00172156 7721353

[26] Amemiya CT Litman GW. Complete Nucleotide Sequence of an Immunoglobulin Heavy-Chain Gene and Analysis of Immunoglobulin Gene Organization in a Primitive Teleost Species. Proc Natl Acad Sci USA (1990) 87:811–5. 10.1073/pnas.87.2.811 PMC53356 2105490

[27] Nakao M Moritomo T Tomana M Fujiki K Yano T. Isolation of cDNA Encoding the Constant Region of the Immuoglobulin Heavy-Chain From Common Carp (*Cyprinus Carpio* L.). Fish Shellfish Immunol (1998) 8:425–34. doi: 10.1006/fsim.1998.0149

[28] FAO. Food and Agriculture Organization of the United Nations. Fishstat Plus Rome (2017).

[29] Srisapoome P Ohira T Hirono I Aoki T. Genes of the Constant Regions of Functional Immunoglobulin Heavy Chain of Japanese Flounder. Paralichthys olivaceus. Immunogen (2004) 56:292–300. doi: 10.1007/s00251-004-0689-7 15243720

[30] Picchietti S Abelli L Buonocore F Randelli E Fausto AM Scapigliati G.. Immunoglobulin Protein and Gene Transcripts in Sea Bream (*Sparus Aurata* L.) Oocytes. Fish Shellfish Immunol (2006) 20:398–404. doi: 10.1016/j.fsi.2005.06.001 16040254

[31] Wilson MR Middleton D Warr GW. Immunoglobulin VH Genes of the Goldfish, *Carassius Auratus*: A Re-Examination. Mol Immunol (1991) 28:449–57. doi: 10.1016/0161-5890(91)90158-G 1905783

[32] Buonocore F Stocchi V Nunez-Ortiz N Randelli E Gerdol M Pallavicini A. Immunoglobulin T From Sea Bass (*Dicentrarchus Labrax* L.): Molecular Characterization, Tissue Localization and Expression After Nodavirus Infection. BMC Mol Biol (2017) 18:8. doi: 10.1186/s12867-017-0085-0 28298204 PMC5353873

[33] Bengten E Stromberg S Pilstrom L. Immunoglobulin VH Regions in Atlantic Cod (*Gadus Morhua* L.): Their Diversity and Relationship to VH Families From Other Species. Dev Comp Immunol (1994) 18:109–22. doi: 10.1016/0145-305X(94)90239-9 8082814

[34] Magadan-Mompo S Sanchez-Espinel C Gambon-Deza F. Immunoglobulin Heavy Chains in Medaka (*Oryzias Latipes*). BMC Evol Biol (2011) 11:165. doi: 10.1186/1471-2148-11-165 21676244 PMC3141427

[35] Hu YL Zhu LY Xiang LX Shao JZ. Discovery of an Unusual Alternative Splicing Pathway of the Immunoglobulin Heavy Chain in a Teleost Fish, *Danio Rerio* . Dev Comp Immunol (2011) 35:253–7. doi: 10.1016/j.dci.2010.10.009 21035505

[36] Waterhouse A Bertoni M Bienert S Studer G Tauriello G Gumienny R. SWISS-MODEL: Homology Modelling of Protein Structures and Complexes. Nucleic Acids Res (2018) 46:W296–303. doi: 10.1093/nar/gky427 PMC6030848 29788355

[37] Karami A Eghtesadi Araghi P Syed MA Wilson SP. Chromosome Preparation in Fish: Effects of Fish Species and Larval Age. Int Aquat Res (2015) 7:201–10. doi: 10.1007/s40071-015-0104-z

[38] Knoll JH Lichter P. *In Situ* Hybridization to Metaphase Chromosomes and Interphase Nuclei. Curr Protoc Hum Genet (2005) 45:4.3.1–4.3.31. doi: 10.1002/0471142905.hg0403s45 18428378

[39] Li S Lefranc MP Miles JJ Alamyar E Giudicelli V Duroux P. IMGT/HighV QUEST Paradigm for T Cell Receptor IMGT Clonotype Diversity and Next Generation Repertoire Immunoprofiling. Nat Commun (2013) 4:2333. doi: 10.1038/ncomms3333 23995877 PMC3778833

[40] Shannon CE. A Mathematical Theory of Communication. Bell Syst Tech (1948) 27:379–423. doi: 10.1002/j.1538-7305.1948.tb01338.x

[41] Kabat EA Wu TT Bilofsky H. Unusual Distributions of Amino Acids in Complementarity-Determining (Hypervariable) Segments of Heavy and Light Chains of Immunoglobulins and Their Possible Roles in Specificity of Antibody-Combining Sites. J Biol Chem (1977) 252:6609–16. doi: 10.1016/S0021-9258(17)39891-5 408353

[42] Chinedu E Arome D Ameh FS. A New Method for Determining Acute Toxicity in Animal Models. Int J Toxicol (2013) 20:224–6. doi: 10.4103/0971-6580.121674 PMC3877490 24403732

[43] Livak KJ Schmittgen TD. Analysis of Relative Gene Expression Data Using Real-Time Quantitative PCR and the 2^(-Delta Delta C(T))^ . Method Methods (San Diego Calif.) (2001) 25:402–8. doi: 10.1006/meth.2001.1262 11846609

[44] Yasuike M de Boer J von Schalburg KR Cooper GA McKinnel L Messmer A. Evolution of Duplicated IgH Loci in Atlantic Salmon. Salmo salar. BMC Genomics (2010) 11:486. doi: 10.1186/1471-2164-11-486 20813058 PMC2996982

[45] Yang F Ventura-Holman T Waldbieser GC Lobb CJ. Structure, Genomic Organization, and Phylogenetic Implications of Six New VH Families in the Channel Catfish. Mol Immunol (2003) 40:247–60. doi: 10.1016/S0161-5890(03)00143-3 12943797

[46] Brown GD Kaattari IM Kaattari SL. Two New Ig VH Gene Families in. Oncorhynchus mykiss. Immunogen (2006) 58:933–6. doi: 10.1007/s00251-006-0149-7 17039360

[47] Phuyindee C Unajak S Srisapoome P. Diversity Analysis of the Immunoglobulin M Heavy Chain Gene in Nile Tilapia, *Oreochromis Niloticus* (Linnaeus). Afr J Biotechnol (2015) 14:2282–99. doi: 10.5897/AJB2014.14001

[48] Andersson E Matsunaga T. Evolutionary Stability of the Immunoglobulin Heavy Chain Variable Region Gene Families in Teleost. Immunogenetics (1998) 47:272–7. doi: 10.1007/s002510050357 9435346

[49] Stenvik J Lundbäck AS Jørgensen TO Pilström L. Variable Region Diversity of the Atlantic Cod (*Gadus Morhua* L.) Immunoglobulin Heavy Chain. Immunogenetics (2000) 51:670–80. doi: 10.1007/s002510000188 10941838

[50] Peixoto BR Brenner S. Characterization of Approximately 50 Kb of the Immunoglobulin VH Locus of the Japanese Pufferfish. Fugu rubripes. Immunogen (2000) 51:443–51. doi: 10.1007/s002510050643 10866111

[51] Rumfelt LL Lohr RL Dooley H Flajnik MF. Diversity and Repertoire of IgW and IgM VH Families in the Newborn Nurse Shark. BMC Immunol (2004) 5:8. doi: 10.1186/1471-2172-5-8 15132758 PMC420240

[52] Lundqvist ML Strömberg S Pilström L. Ig Heavy Chain of the Sturgeon *Acipenser Baeri*: cDNA Sequence and Diversity. Immunogenetics (1998) 48:372–82. doi: 10.1007/s002510050448 9799333

[53] Haire RN Amemiya CT Suzuki D Litman GW. Eleven Distinct VH Gene Families and Additional Patterns of Sequence Variation Suggest a High Degree of Immunoglobulin Gene Complexity in a Lower Vertebrate. Xenopus laevis. J Exp Med (1990) 171:1721–37. doi: 10.1084/jem.171.5.1721 PMC2187900 2110243

[54] Ota T Nei M. Evolution of Immunoglobulin VH Pseudogenes in Chickens. Mol Biol Evol (1995) 12:94–102. doi: 10.1093/oxfordjournals.molbev.a040194 7877500

[55] Mainville CA Sheehan KM Klaman LD Giorgetti CA Press JL Brodeur PH. Deletional Mapping of Fifteen Mouse VH Gene Families Reveals a Common Organization for Three IgH Haplotypes. J Immunol (1996) 156:1038–46. 8557977

[56] Pinheiro A Lanning D Alves PC Mage RG Knight KL van der Loo W. Molecular Bases of Genetic Diversity and Evolution of the Immunoglobulin Heavy Chain Variable Region (IGHV) Gene Locus in Leporids. Immunogenetics (2011) 63:397–408. doi: 10.1007/s00251-011-0533-9 21594770 PMC3404511

[57] Sun J Kacskovics I Brown WR Butler JE. Expressed Swine VH Genes Belong to a Small VH Gene Family Homologous to Human VHIII. J Immunol (1994) 153:5618–27. 7989761

[58] Matsuda F Ishii K Bourvagnet P Kuma KiHayashida H Miyata T. The Complete Nucleotide Sequence of the Human Immunoglobulin Heavy Chain Variable Region Locus. J Exp Med (1998) 188:2151–62. doi: 10.1084/jem.188.11.2151 PMC2212390 9841928

[59] Lefranc MP Giudicelli V Kaas Q Duprat E Jabado-Michaloud J Scaviner D. IMGT, the International ImMunoGeneTics Information System. Nucleic Acids Res (2005) 33:D593–7. doi: 10.1093/nar/gki065 PMC540019 15608269

[60] Lefranc MP Giudicelli V Ginestoux C Bodmer J Müller W Bontrop R. IMGT, the International ImMunoGeneTics Database. Nucleic Acids Res (1999) 27:209–12. doi: 10.1093/nar/27.1.209 PMC148137 9847182

[61] Hansen JD Landis ED Phillips RB. Discovery of a Unique Ig Heavy-Chain Isotype (IgT) in Rainbow Trout: Implications for a Distinctive B Cell Developmental Pathway in Teleost Fish. Proc Natl Acad Sci USA (2005) 102:6919–24. doi: 10.1073/pnas.0500027102 PMC1100771 15863615

[62] Fillatreau S Six A Magadan S Castro R Sunyer JO Boudinot P. The Astonishing Diversity of Ig Classes and B Cell Repertoires in Teleost Fish. Front Immunol (2013) 4. doi: 10.3389/fimmu.2013.00028 PMC3570791 23408183

[63] Bengtén E Quiniou SM Stuge TB Katagiri T Miller NW Clem LW. The IgH Locus of the Channel Catfish, *Ictalurus Punctatus*, Contains Multiple Constant Region Gene Dequences: Different Genes Encode Heavy Chains of Membrane and Secreted IgD. J Immunol (2002) 169:2488. doi: 10.4049/jimmunol.169.5.2488 12193718

[64] Bruce SR Dingle RWC. Peterson Ml. B-cell Plasma-Cell Splicing Differences: A Potential Role Regulated Immunoglobulin RNA Processing. RNA (2003) 9:1264–73. doi: 10.1261/rna.5820103 PMC1370490 13130140

[65] Ventura-Holman T Lobb CJ. Structural Organization of the Immunoglobulin Heavy Chain Locus in the Channel Catfish: The IgH Locus Represents a Composite of Two Gene Clusters. Mol Immunol (2002) 38:557–64. doi: 10.1016/S0161-5890(01)00075-X 11750657

[66] Danilova N Saunders HL Ellestad KK Magor BG. The Zebrafish IgH Locus Contains Multiple Transcriptional Regulatory Regions. Dev Comp Immunol (2011) 35:352–9. doi: 10.1016/j.dci.2010.10.010 PMC3031712 21055416

[67] Schwager J Mikoryak CA Steiner LA. Amino Acid Sequence of Heavy Chain From *Xenopus Laevis* IgM Deduced From cDNA Sequence: Implications for Evolution of Immunoglobulin Domains. Proc Natl Acad Sci USA (1988) 85:2245–9. doi: 10.1073/pnas.85.7.2245 PMC279967 2451244

[68] Schwager J Grossberger D Du Pasquier L. Organization and Rearrangement of Immunoglobulin M Genes in the Amphibian Xenopus. EMBO J 1988 7(8):2409–15. doi: 10.1002/j.1460-2075.1988.tb03086.x PMC457108 2903824

[69] Lee V Huang JL Lui MF Malecek K Ohta Y Mooers A. The Evolution of Multiple Isotypic IgM Heavy Chain Genes in the Shark. J Immunol (2008) 180:7461–70. doi: 10.4049/jimmunol.180.11.7461 PMC2590587 18490746

[70] McGuire KL Duncan WR Tucker PW. Phylogenetic Conservation of Immunoglobulin Heavy Chains: Direct Comparison of Hamster and Mouse Cμ Genes. Nucleic Acids Res (1985) 13:5611–28. doi: 10.1093/nar/13.15.5611 PMC321893 2994005

[71] Early P Rogers J Davis M Calame K Bond M Wall R. Two mRNAs Can Be Produced From a Single Immunoglobulin μ Gene by Alternative RNA Processing Pathways. Cell (1980) 20:313–9. doi: 10.1016/0092-8674(80)90617-0 6771020

[72] Wilson MR Marcuz A van Ginkel F Miller NW Clem LW Middleton D. The Immunoglobulin M Heavy Chain Constant Region Gene of the Channel Catfish, *Ictalurus Punctatus*: An Unusual mRNA Splice Pattern Produces the Membrane Form of the Molecule. Nucleic Acids Res (1990) 18:5227–33. doi: 10.1093/nar/18.17.5227 PMC332146 2119496

[73] Ghaffari SH Lobb CJ. Nucleotide Sequence of Channel Catfish Heavy Chain cDNA and Genomic Blot Analyses. Implications Phylogeny Ig Heavy Chains. J Immunol (1989) 143:2730–9. 2507636

[74] Lee MA Bengtén E Daggfeldt A Rytting A-S Pilström L. Characterisation of Rainbow Trout cDNAs Encoding a Secreted and Membrane-Bound Ig Heavy Chain and the Genomic Intron Upstream of the First Constant Exon. Mol Immunol (1993) 30:641–8. doi: 10.1016/0161-5890(93)90075-M 8487781

[75] Harris LJ Larson SB McPherson A. Comparison of Intact Antibody Structures and the Implications for Effector Function. Adv Immunol (1999) 72:191–208. doi: 10.1016/S0065-2776(08)60021-8 10361576

[76] Andersson E Matsunaga T. Evolution of Immunoglobulin Heavy Chain Variable Region Genes: A VH Family Can Last for 150-200 Million Years or Longer. Immunogenetics (1995) 41:18–28. doi: 10.1007/BF00188428 7806270

[77] Roman T De Guerra A Charlemagne J. Evolution of Specific Antigen Recognition: Size Reduction and Restricted Length Distribution of the CDRH3 Regions in the Rainbow Trout. Eur J Immunol (1995) 25:269–73. doi: 10.1002/eji.1830250144 7843242

[78] Brodeur PH Riblet R. The Immunoglobulin Heavy Chain Variable Region (Igh-V) Locus in the Mouse. I. One Hundred Igh-V Genes Comprise Seven Families Homologous Genes. Eur J Immunol (1984) 14:922–30. doi: 10.1002/eji.1830141012 6092095

[79] Stenvik J Schrøder MB Olsen K Zapata A Jørgensen TØ. Expression of Immunoglobulin Heavy Chain Transcripts (VH-Families, IgM, and IgD) in Head Kidney and Spleen of the Atlantic Cod (*Gadus Morhua* L.). Dev Comp Immunol (2001) 25:291–302. doi: 10.1016/S0145-305X(00)00056-2 11246069

