# Supplementary material for: Mystifying Molecular Structure, Expression and Repertoire Diversity of IgM Heavy Chain Genes (Ighμ) in Clarias Catfish and Hybrids: Two Novel Transcripts in Vertebrates
Source: Front Immunol. 2022 Jun 17;13:884434. doi: 10.3389/fimmu.2022.884434 (PMC9247300; doi:10.3389/fimmu.2022.884434)
Supplement: Supplementary file 1 [file DataSheet_1.pdf]

```
        Cm_ighμ1
   1  GCAACACAAGAAGCCCCGAAGTCCCTGTTTCCCGTGTGGCAGTGCGGCTCGTCCCCTGAT  60
       A  T  Q  E  A  P  K  S  L  F  P  V  W  Q  C  G  S  S  P  D

  61  GGTTTTGTCACTCTGGGCTGCGTCACCCGCGACTTGGCAACCCCTGATGGACTGAGCTAC  120
       G  F  V  T  L  G  C  V  T  R  D  L  A  T  P  D  G  L  S  Y

 121  GTTTGGAAGGATGCGAGCGGCACCGCGCTGACTACTAACGTTGTACAATACCCGGCGGTG  180
       V  W  K  D  A  S  G  T  A  L  T  T  N  V  V  Q  Y  P  A  V

 181  ATCGAGGGCGGAAAGTACTCCTCAGTGAGTCAGGCGCGCGTCAGTGCTAACGACTGGAAC  240
       I  E  G  G  K  Y  S  S  V  S  Q  A  R  V  S  A  N  D  W  N

 241  GCGAACAAGAAGTTCACTTGCGAAGTCACTAATCCCCGGGGAACGAAAACGGCAGAGCTG  300
       A  N  K  K  F  T  C  E  V  T  N  P  R  G  T  K  T  A  E  L

                       Cm_ighμ2
 301  AAAAAGCCAGTTGTGCCCGACATCCCTGCAAAATCTCTGCTTCTAACAGCTCCGAGCCAA  360
       K  K  P  V  V  P  D  I  P  A  K  S  L  L  L  T  A  P  S  Q

 361  ACAGAATTAGAAAATGGAACAGCTACCTTCATCTGCTTAGCTTCAGAATTTTCACCTAAA  420
       T  E  L  E  N  G  T  A  T  F  I  C  L  A  S  E  F  S  P  K

 421  ACGTCCACATTTAAGTGGACCCGTGGAGGAACAAGCATCGATAATAAGGCCAAAGACCCC  480
       T  S  T  F  K  W  T  R  G  G  T  S  I  D  N  K  A  K  D  P

 481  ATTTTAATCGCGGGAAAACCTACTTACAGTTCCTTTAGTATTTTGGTACTCACTGCTGCC  540
       I  L  I  A  G  K  P  T  Y  S  S  F  S  I  L  V  L  T  A  A

 541  GAATGGACTGGCTCAACTTCTCCAGTTAAGTGCGAGTTCCAGCAGAAGTCACAGACTCTG  600
       E  W  T  G  S  T  S  P  V  K  C  E  F  Q  Q  K  S  Q  T  L

                                   Cm_ighμ3
 601  TCCAAGGAGGCAAGTTATCATTATGTGACCCGGGAGCAGCCAACAATTACAATAATCCCA  660
       S  K  E  A  S  Y  H  Y  V  T  R  E  Q  P  T  I  T  I  I  P

 661  CCTTCCAACAATGACATGCTTATCAAAAGATCTGGCGACCTCGTGTGCAAGGCTGAAGGA  720
       P  S  N  N  D  M  L  I  K  R  S  G  D  L  V  C  K  A  E  G

 721  CCATTGGGTTTCACTGGAATTAAGTGGGTTGCTAACGGTAAAGAGGTTGTTTCCCTATCT  780
       P  L  G  F  T  G  I  K  W  V  A  N  G  K  E  V  V  S  L  S

 781  GAAAAAGTTGTGTCTACGAAGGCAGCGATCTCACTCACCACCTCAATCTCTTACGAAGAG  840
       E  K  V  V  S  T  K  A  A  I  S  L  T  T  S  I  S  Y  E  E

 841  TGGCATAAGGGCATGAAATTTACCTGTGAGGTGCATCATTCCTCATTTGCTCAGGGGTTT  900
       W  H  K  G  M  K  F  T  C  E  V  H  H  S  S  F  A  Q  G  F

                                             Cm_ighμtm1
 901  ATAACAGAAGTCTACAAAAGAGAAAATTATGCCTTAACATTTGTATTTCTCTTCCTCATC  960
       I  T  E  V  Y  K  R  E  N  Y  A  L  T  F  V  F  L  F  L  I

                                             Cm_ighμtm2
 961  ACCCTGTTCTACAGCATCGGGGTGACTGTTATCAAGGTGAAATGGGAAACAACTAATGGA  1020
       T  L  F  Y  S  I  G  V  T  V  I  K  V  K  W  E  T  T  N  G

1021  ACGATTTTGATTTTCTCTCTGTTTTTTTTTTCTTCAGTATAAGTTTGTTTGTTTTGTTTT  1080
       T  I  L  I  F  S  L  F  F  F  S  S  V  *

1081  TTTTATTTTAGTGTTCTGAGTTACCCTTTCTGTATCATCTAGAAGATCTCTACATCACGG  1140
1141  CAAATACTTTTGGACATTTAAATGTTGGACATTTTTTTCATGCATTTACTCAAATCTCAA  1200
1201  ATAAATGTCTTACAAAGTATGCAAATAAAAAAAAAAAAAAAAAAAAAAAAAAAAAAAAAA  1260
1261  AAA                                                           1263
```

Supplementary Fig. 1. Nucleotide and translated amino acid sequences of the membrane-bound IgM constant domain of the bighead catfish (*Cm*_mIgM 1.1, GenBank accession number MN934742) and hybrid catfish (hybrid:*Cm*_mIgM 1.1, GenBank accession number MN934751). The sequence was consisted of three CH and two TM domains: Cμ1-Cμ2-Cμ3-TM1-TM2. The atypical polyadenylation signal (AATTAAA) in the 3′-UTR was bold.

→Cm_ighμ1

1 GCAACACAAGAAGCCCCGAAGTCCCTGTTTCCCGTGTGGCAGTGCGGCTCGTCCCCTGAT 60
A T Q E A P K S L F P V W Q C G S S P D

61 GGTTTTGTCACTCTGGGCTGCGTCACCCGCGACTTGGCAACCCCTGATGGACTGAGCTAC 120
G F V T L G C V T R D L A T P D G L S Y

121 GTTTGGAAGGATGCGAGCGGCACCGCGCTGACTACTAACGTTGTACAATACCCGGCGGTG 180
V W K D A S G T A L T T N V V Q Y P A V

181 ATCGAGGGCGGAAAGTACTCCTCAGTGAGTCAGGCGCGCGTCAGTGCTAACGACTGGAAC 240
I E G G K Y S S V S Q A R V S A N D W N

241 GCGAACAAGAAGTTCACTTGCGAAGTCACTAATCCCCGGGGAACGAAAACGGCAGAGCTG 300
A N K K F T C E V T N P R G T K T A E L

→Cm_ighμ2

301 AAAAAGCCAGTTGTGCCCGACATCCCTGCAAAATCTCTGCTTCTAACAGCTCCGAGCCAA 360
K K P V V P D I P A K S L L L T A P S Q

361 ACAGAATTAGAAAATGGAACAGCTACCTTCATCTGCTTAGCTTCAGAATTTTCACCTAAA 420
T E L E N G T A T F I C L A S E F S P K

421 ACGTCCACATTTAAGTGGACCCGTGGAGGAACAAGCATCGATAATAAGGCCAAAGACCCC 480
T S T F K W T R G G T S I D N K A K D P

481 ATTTTAATCGCGGGAAAACCTACTTACAGTTCCTTTAGTATTTTGGTACTCACTGCTGCC 540
I L I A G K P T Y S S F S I L V L T A A

541 GAATGGACTGGCTCAACTTCTCCAGTTAAGTGCGAGTTCCAGCAGAAGTCACAGACTCTG 600
E W T G S T S P V K C E F Q Q K S Q T L

→Cm_ighμ3

601 TCCAAGGAGGCAAGTTATCATTATGTGACCCGGGAGCAGCCAACAATTACAATAATCCCA 660
S K E A S Y H Y V T R E Q P T I T I I P

661 CCTTCCAACAATGACATGCTTATCAAAAGATCTGGCGACCTCGTGTGCAAGGCTGAAGGA 720
P S N N D M L I K R S G D L V C K A E G

721 CCATTGGGTTTCACTGGAATTAAGTGGGTTGCTAACGGTAAAGAGGTTGTTTCCCTATCT 780
P L G F T G I K W V A N G K E V V S L S

781 GAAAAAGTTGTGTCTACGAAGGCAGCGATCTCACTCACCACCTCAATCTCTTACGAAGAG 840
E K V V S T K A A I S L T T S I S Y E E

841 TGGCATAAGGGCATGAAATTTACCTGTGAGGTGCATCATTCCTCATTTGCTCAGGGGTTT 900
W H K G M K F T C E V H H S S F A Q G F

→Cm_ighμtm1

901 ATAACAGAAGTCTACAAAAGAGAAAATTATGCCTTAACATTTGTATTTCTCTTCCTCATC 960
I T E V Y K R E N Y A L T F V F L F L I

→Cm_ighμtm2

961 ACCCTGTTCTACAGCATCGGGGTGACTGTTATCAAGGTGAAATGGGAAACAACTAATGGA 1020
T L F Y S I G V T V I K V K W E T T N G

1021 ACGATTTTGATTTTCTCTCTGTTTTTTTTTTCTTCAGTATAAGTTTGTTTGTTTTGTTTT 1080
T I L I F S L F F F S S V *

1081 TTTTATTTTAGTGTTCTGAGTTACCCTTTCTGTATCATCTAGAAGATCTCTACATCACGG 1140
1141 CAAATACTTTTGGACATTTAAATGTTGGACATTTTTTTCATGCATTTACTCAAATCTCAA 1200
1201 ATAAATATTTGACAAAAATATGCAATTAAAAGTCTAATTGTTTTCTGTATCTTTATTTGA 1260
1261 ATTATAGTCAGCCTTAAAGTATAGTGTTTATGTTATTAGCTTATATTACATGTCTATATT 1320
1321 TTATTAGTTTTT**AATAAA**TGTCTTACAAAAATATGCGAAAAAAAAAAAAAAAAAAAAAA 1380
1381 AAAAAAA 1387

Supplementary Fig. 2. Nucleotide and translated amino acid sequences of the membrane-bound IgM constant domain of the bighead catfish (*Cm*_mIgM 1.2, GenBank accession number MN934743). The sequence was consisted of three CH and two TM domains: Cμ1-Cμ2-Cμ3-TM1-TM2. The atypical polyadenylation signal (AATTAAA) in the 3′-UTR was bold.

```
       ┌→ Cm_ighμ1
   1   GCAACACAAGAAGCCCCGAAGTCCCTGTTTCCCGTGTGGCAGTGCGGCTCGTCCCCTGAT   60
        A  T  Q  E  A  P  K  S  L  F  P  V  W  Q  C  G  S  S  P  D

  61   GGTTTTGTCACTCTGGGCTGCGTCACCCGCGACTTGGCAACCCCTGATGGACTGAGCTAC   120
        G  F  V  T  L  G  C  V  T  R  D  L  A  T  P  D  G  L  S  Y

 121   GTTTGGAAGGATGCGAGCGGCACCGCGCTGACTACTAACGTTGTACAATACCCGGCGGTG   180
        V  W  K  D  A  S  G  T  A  L  T  T  N  V  V  Q  Y  P  A  V

 181   ATCGAGGGCGGAAAGTACTCCTCAGTGAGTCAGGCGCGCGTCAGTGCTAACGACTGGAAC   240
        I  E  G  G  K  Y  S  S  V  S  Q  A  R  V  S  A  N  D  W  N

 241   GCGAACAAGAAGTTCACTTGCGAAGTCACTAATCCCCGGGGAACGAAAACGGCAGAGCTG   300
        A  N  K  K  F  T  C  E  V  T  N  P  R  G  T  K  T  A  E  L

                ┌→ Cm_ighμ2
 301   AAAAAGCCAGTTGTGCCCGACATCCCTGCAAAATCTCTGCTTCTAACAGCTCCGAGCCAA   360
        K  K  P  V  V  P  D  I  P  A  K  S  L  L  L  T  A  P  S  Q

 361   ACAGAATTAGAAAATGGAACAGCTACCTTCATCTGCTTAGCTTCAGAATTTTCACCTAAA   420
        T  E  L  E  N  G  T  A  T  F  I  C  L  A  S  E  F  S  P  K

 421   ACGTCCACATTTAAGTGGACCCGTGGAGGAACAAGCATCGATAATAAGGCCAAAGACCCC   480
        T  S  T  F  K  W  T  R  G  G  T  S  I  D  N  K  A  K  D  P

 481   ATTTTAATCGCGGGAAAACCTACTTACAGTTCCTTTAGTATTTTGGTACTCACTGCTGCC   540
        I  L  I  A  G  K  P  T  Y  S  S  F  S  I  L  V  L  T  A  A

 541   GAATGGACTGGCTCAACTTCTCCAGTTAAGTGCGAGTTCCAGCAGAAGTCACAGACTCTG   600
        E  W  T  G  S  T  S  P  V  K  C  E  F  Q  Q  K  S  Q  T  L

                         ┌→ Cm_ighμ3
 601   TCCAAGGAGGCAAGTTATCATTATGTGACCCGGGAGCAGCCAACAATTACAATAATCCCA   660
        S  K  E  A  S  Y  H  Y  V  T  R  E  Q  P  T  I  T  I  I  P

 661   CCTTCCAACAATGACATGCTTATCAAAAGATCTGGCGACCTCGTGTGCAAGGCTGAAGGA   720
        P  S  N  N  D  M  L  I  K  R  S  G  D  L  V  C  K  A  E  G

 721   CCATTGGGTTTCACTGGAATTAAGTGGGTTGCTAACGGTAAAGAGGTTGTTTCCCTATCT   780
        P  L  G  F  T  G  I  K  W  V  A  N  G  K  E  V  V  S  L  S

 781   GAAAAAGTTGTGTCTACGAAGGCAGCGATCTCACTCACCACCTCAATCTCTTACGAAGAG   840
        E  K  V  V  S  T  K  A  A  I  S  L  T  T  S  I  S  Y  E  E

 841   TGGCATAAGGGCATGAAATTTACCTGTGAGGTGCATCATTCCTCATTTGCTCAGGGGTTT   900
        W  H  K  G  M  K  F  T  C  E  V  H  H  S  S  F  A  Q  G  F

                                    ┌→ Cm_ighμtm1
 901   ATAACAGAAGTCTACAAAAGAGAAAATTATGCCTTAACATTTGTATTTCTCTTCCTCATC   960
        I  T  E  V  Y  K  R  E  N  Y  A  L  T  F  V  F  L  F  L  I

                                    ┌→ Cm_ighμtm2
 961   ACCCTGTTCTACAGCATCGGGGTGACTGTTATCAAGGTGAAATGGGAAACAACTAATGGA   1020
        T  L  F  Y  S  I  G  V  T  V  I  K  V  K  W  E  T  T  N  G

1021   ACGATTTTGATTTTCTCTCTGTTTTTTTTTTCTTCAGTATAAGTTTGTTTGTTTTGTTTT   1080
        T  I  L  I  F  S  L  F  F  F  S  S  V  *

1081   TTTTATTTTAGTGTTCTGAGTTACCCTTTCTGTATCATCTAGAAGATCTCTACATCACGG   1140
1141   CAAATACTTTTGGACATTTAAATGTTGGACATTTTTTTCATGCATTTACTCAAATCTCAA   1200
1201   ATAAATATTTGACAAAAATATGCAATTAAAAGTCTAATTGTTTTCTGTATCTTTATTTGA   1260
1261   ATTATAGTCAGCCTTAAAGTATAGTGTTTATGTTATTAGCTTATATTACATGTCTATATT   1320
1321   TTATTAGTTTTTAATAAAATTATTAAGACGTTTCGATGACAGTAGTGTTTCTCTCAGTTT   1380
1381   GCTGTTGAAACATAGTAAAACTATTGAGTTATATTATTTATTGATCTTTTTAGCTAATAG   1440
1441   ATGTAGAGATCCTGGTGCTGACCAACCTTTTCTACAATGAGACAGAACTTGATGATGACA   1500
1501   ACGTGGCAACCACTGCCTTAACATTTGTATTTCTCTTCCTCATCACCCTGTTCTACAGCA   1560
1561   TCGGGGTGACTGTTATCAAGGTGAAATGAGAAACAATAAATGCAATTAAAAGTCAAAAAA   1620
1621   AAAAAAAAAAAAAAAAAAAAA                                          1641
```

Supplementary Fig. 3. Nucleotide and translated amino acid sequences of the membrane-bound IgM constant domain of the bighead catfish (*Cm*_mIgM 1.3, GenBank accession number MN934744). The sequence was consisted of three CH and two TM domains: Cμ1-Cμ2-Cμ3-TM1-TM2. The atypical polyadenylation signal (AATTAAA) in the 3′-UTR was bold.

```
     →Cm_ighμ1
1    GCAACACAAGAAGCCCCGAAGTCCCTGTTTCCCGTGTGGCAGTGCGGCTCGTCCCCTGAT  60
      A  T  Q  E  A  P  K  S  L  F  P  V  W  Q  C  G  S  S  P  D

61   GGTTTTGTCACTCTGGGCTGCGTCACCCGCGACTTGGCAACCCCTGATGGACTGAGCTAC  120
      G  F  V  T  L  G  C  V  T  R  D  L  A  T  P  D  G  L  S  Y

121  GTTTGGAAGGATGCGAGCGGCACCGCGCTGACTACTAACGTTGTACAATACCCGGCGGTG  180
      V  W  K  D  A  S  G  T  A  L  T  T  N  V  V  Q  Y  P  A  V

181  ATCGAGGGCGGAAAGTACTCCTCAGTGAGTCAGGCGCGCGTCAGTGCTAACGACTGGAAC  240
      I  E  G  G  K  Y  S  S  V  S  Q  A  R  V  S  A  N  D  W  N

241  GCGAACAAGAAGTTCACTTGCGAAGTCACTAATCCCCGGGGAACGAAAACGGCAGAGCTG  300
      A  N  K  K  F  T  C  E  V  T  N  P  R  G  T  K  T  A  E  L
                  →Cm_ighμ2
301  AAAAAGCCAGTTGTGCCCGACATCCCTGCAAAATCTCTGCTTCTAACAGCTCCGAGCCAA  360
      K  K  P  V  V  P  D  I  P  A  K  S  L  L  L  T  A  P  S  Q

361  ACAGAATTAGAAAATGGAACAGCTACCTTCATCTGCTTAGCTTCAGAATTTTCACCTAAA  420
      T  E  L  E  N  G  T  A  T  F  I  C  L  A  S  E  F  S  P  K

421  ACGTCCACATTTAAGTGGACCCGTGGAGGAACAAGCATCGATAATAAGGCCAAAGACCCC  480
      T  S  T  F  K  W  T  R  G  G  T  S  I  D  N  K  A  K  D  P

481  ATTTTAATCGCGGGAAAACCTACTTACAGTTCCTTTAGTATTTTGGTACTCACTGCTGCC  540
      I  L  I  A  G  K  P  T  Y  S  S  F  S  I  L  V  L  T  A  A

541  GAATGGACTGGCTCAACTTCTCCAGTTAAGTGCGAGTTCCAGCAGAAGTCACAGACTCTG  600
      E  W  T  G  S  T  S  P  V  K  C  E  F  Q  Q  K  S  Q  T  L
                        →Cm_ighμ3
601  TCCAAGGAGGCAAGTTATCATTATGTGACCCGGGAGCAGCCAACAATTACAATAATCCCA  660
      S  K  E  A  S  Y  H  Y  V  T  R  E  Q  P  T  I  T  I  I  P

661  CCTTCCAACAATGACATGCTTATCAAAAGATCTGGCGACCTCGTGTGCAAGGCTGAAGGA  720
      P  S  N  N  D  M  L  I  K  R  S  G  D  L  V  C  K  A  E  G

721  CCATTGGGTTTCACTGGAATTAAGTGGGTTGCTAACGGTAAAGAGGTTGTTTCCCTATCT  780
      P  L  G  F  T  G  I  K  W  V  A  N  G  K  E  V  V  S  L  S

781  GAAAAAGTTGTGTCTACGAAGGCAGCGATCTCACTCACCACCTCAATCTCTTACGAAGAG  840
      E  K  V  V  S  T  K  A  A  I  S  L  T  T  S  I  S  Y  E  E

841  TGGCATAAGGGCATGAAATTTACCTGTGAGGTGCATCATTCCTCATTTGCTCAGGGGTTT  900
      W  H  K  G  M  K  F  T  C  E  V  H  H  S  S  F  A  Q  G  F
                                       →Cm_ighμ4
901  ATAACAGAAGTCTACAAAAGAGAAAATGGCCGAACGCCTAACTGCCCAGGGGTTTTCTTG  960
      I  T  E  V  Y  K  R  E  N  G  R  T  P  N  C  P  G  V  F  L

961  CTCCCGCCACCAGAGAGCTCAAATGGGGATTTGAAGACCCTGACTTGCTATATAAAAAAC  1020
      L  P  P  P  E  S  S  N  G  D  L  K  T  L  T  C  Y  I  K  N

1021 TTTTACCCTAAGGAGGTTGCTGTGTCTTGGCTTATTGGTGATAAACAAGTGGACAATGAG  1080
      F  Y  P  K  E  V  A  V  S  W  L  I  G  D  K  Q  V  D  N  E

1081 AGCATCACTAAAGTTATTGAGAAAAATGGCAACTTTTCAGCATACAGTCAGCTGATTGTC  1140
      S  I  T  K  V  I  E  K  N  G  N  F  S  A  Y  S  Q  L  I  V

1141 AATCAGGACTCCTGGGGAAATGGCACAGAGTTCACCTGCAATGTTTATCATGAGTCCATC  1200
      N  Q  D  S  W  G  N  G  T  E  F  T  C  N  V  Y  H  E  S  I

1201 ATGGATAATCTTCGCCACCTTTCCAGAACCATTACTGGCACTTCAAATCCACCCTCCATA  1260
      M  D  N  L  R  H  L  S  R  T  I  T  G  T  S  N  P  P  S  I
                                                           →Cm_ighμtm1
1261 GTGAATCTCAGCCTAAATGTTCCCCAAAACTGCCCAAATCCTGGTCTGTATGCCTTAACA  1320
      V  N  L  S  L  N  V  P  Q  N  C  P  N  P  G  L  Y  A  L  T
                                                           →Cm_ighμtm2
1321 TTTGTATTTCTCTTCCTCATCACCCTGTTCTACAGCATCGGGGTGACTGTTATCAAGGTG  1380
      F  V  F  L  F  L  I  T  L  F  Y  S  I  G  V  T  V  I  K  V

1381 AAATGGGAAACAACTAATGGAACGATTTTGATTTTCTCTCTGTTTTTTTTTTCTTCAGTA  1440
      K  W  E  T  T  N  G  T  I  L  I  F  S  L  F  F  F  S  S  V

1441 TAAGTTTGTTTGTTTTGTTTTTTTTATTTTAGTGTTCTGAGTTACCCTTTCTGTATCATC  1500
      *
1501 TAGAAGATCTCTACATCACGGCAAATACTTTTGGACATTTAAATGTTGGACATTTTTTTC  1560
1561 ATGCATTTACTCAAATCTCAAATAAATATTTGACAAAAATATGCAATTAAAAGTCTAATT  1620
1621 GTTTTCTGTATCTTTATTTGAATTATAGTCAGCCTTAAAGTATAGTGTTTATGTTATTAG  1680
1681 CTTATATTACATGTCTATATTTTATTAGTTTTTAATAAAATTATTAAGACGTTTCGATGA  1740
1741 CAGTAGTGTTTCTCTCAGTTTGCTGTTGAAACATAGTAAAACTATTGAGTTATATTATTT  1800
1801 ATTGATCTTTTTAGCTAATAGATGTAGAGATCCTGGTGCTGACCAACCTTTTCTACAATG  1860
1861 AGACAGAACTTGATGATGACAACGTGGCAACCACTGCCTTAACATTTGTATTTCTCTTCC  1920
1921 TCATCACCCTGTTCTACAGCATCGGGGTGACTGTTATCAAGGTGAAATGAGAAACAATAA  1980
1981 ATGCAATTAAAAGTCAAAAAAAAAAAAAAAAAAAAAAAAAAAAA  2022
```

Supplementary Fig. 4. Nucleotide and translated amino acid sequences of the membrane-bound IgM constant domain of the bighead catfish (*Cm*_mIgM 2, GenBank accession number MN934745). The sequence was consisted of three CH and two TM domains: Cμ1-Cμ2-Cμ3-Cμ4-TM1-TM2. The atypical polyadenylation signal (AATTAAA) in the 3′-UTR was bold.

```
        ┌→Cm_ighμ1
   1    GCAACACAAGAAGCCCCGAAGTCCCTGTTTCCCGTGTGGCAGTGCGGCTCGTCCCCTGAT   60
         A  T  Q  E  A  P  K  S  L  F  P  V  W  Q  C  G  S  S  P  D

  61    GGTTTTGTCACTCTGGGCTGCGTCACCCGCGACTTGGCAACCCCTGATGGACTGAGCTAC   120
         G  F  V  T  L  G  C  V  T  R  D  L  A  T  P  D  G  L  S  Y

 121    GTTTGGAAGGATGCGAGCGGCACCGCGCTGACTACTAACGTTGTACAATACCCGGCGGTG   180
         V  W  K  D  A  S  G  T  A  L  T  T  N  V  V  Q  Y  P  A  V

 181    ATCGAGGGCGGAAAGTACTCCTCAGTGAGTCAGGCGCGCGTCAGTGCTAACGACTGGAAC   240
         I  E  G  G  K  Y  S  S  V  S  Q  A  R  V  S  A  N  D  W  N

 241    GCGAACAAGAAGTTCACTTGCGAAGTCACTAATCCCCGGGGAACGAAAACGGCAGAGCTG   300
         A  N  K  K  F  T  C  E  V  T  N  P  R  G  T  K  T  A  E  L

                 ┌→Cm_ighδ2
 301    AAAAAGCCAGAAGTGAATGTGACTTCGCCCAGCATCAGCATCAGCACAAGCTTCGACACA   360
         K  K  P  E  V  N  V  T  S  P  S  I  S  I  S  T  S  F  D  T

 361    ACAACTAACGATACTAATCTGATTTGCTGGTTGGATGGTTTTAGCCCTAAAGCGATTAAT   420
         T  T  N  D  T  N  L  I  C  W  L  D  G  F  S  P  K  A  I  N

 421    GTTACTTGGGGAGCGGGTAAGAAAGGGACTGAGAGAAAATTCAAAAATGAGGAAAATAAA   480
         V  T  W  G  A  G  K  K  G  T  E  R  K  F  K  N  E  E  N  K

 481    AAGTTTGCCATGCTGAGCCAAATCAGCATAAATGCAGAGCAATGGAAAGAAGGCAAAGAG   540
         K  F  A  M  L  S  Q  I  S  I  N  A  E  Q  W  K  E  G  K  E

                                          ┌→Cm_ighδ3
 541    TTCACCTGCAGCGCCACACACAAGTCTAAAACCTACAGTCAAACATGGAGCATTTGTAAA   600
         F  T  C  S  A  T  H  K  S  K  T  Y  S  Q  T  W  S  I  C  K

 601    GGCCATCTAAATTCTGAACCACGGATACGTCTGGAGAAACCTGCTCTCAGGTCCATATTG   660
         G  H  L  N  S  E  P  R  I  R  L  E  K  P  A  L  R  S  I  L

 661    ACAGATACACGTGTAACAGTCTCCTGTGTTGTTGAAACTGTGTTCCAGCCTAAAGTTTTA   720
         T  D  T  R  V  T  V  S  C  V  V  E  T  V  F  Q  P  K  V  L

 721    TGGCTTGTAGATGGAAGCACAAAATCTGACACAGACATTATCAGTGAGAGTCAAGGCGAG   780
         W  L  V  D  G  S  T  K  S  D  T  D  I  I  S  E  S  Q  G  E

 781    TCCACTGTTAGCAAACTGACTATTTCTGTAGAAGACTGGACAAAATCAAACACAATAACC   840
         S  T  V  S  K  L  T  I  S  V  E  D  W  T  K  S  N  T  I  T

 841    TGCAGGGCCGAACATCCATTGTTGAGATCTTCTAGATGTTCATTCAAAACTACAGAAACT   900
         C  R  A  E  H  P  L  L  R  S  S  R  C  S  F  K  T  T  E  T

 901    GTGCAGAAACGTCCTACAGTGGAGATCAGAAGGAGTCTTGAAGACGTAGGGAAGAGAGAC   960
         V  Q  K  R  P  T  V  E  I  R  R  S  L  E  D  V  G  K  R  D

 961    AGTGCAGTGCTGGAGTGTGCTGCAAGCGGTCTGCCTCCTGGTGAACTCTCTGTCACCTTC   1020
         S  A  V  L  E  C  A  A  S  G  L  P  P  G  E  L  S  V  T  F

1021    CAGGCCAATAATGTGAATTTTCCAGAAGCTCAGTATGTTAATCTACCAAAAGGCCAGGAC   1080
         Q  A  N  N  V  N  F  P  E  A  Q  Y  V  N  L  P  K  G  Q  D

1081    ACACTAGTTGCACTTTTCACTATTCCTGGAACACACCGAACCGAACAGCATCGCTTCACT   1140
         T  L  V  A  L  F  T  I  P  G  T  H  R  T  E  Q  H  R  F  T

1141    TGTGAGATTCAGACAAGCCGTTCCTTAAAATGGAGGTCCAATTTTATAGGAAACCTTTTT   1200
         C  E  I  Q  T  S  R  S  L  K  W  R  S  N  F  I  G  N  L  F

                 ┌→Cm_ighδ4
1201    AGTGATCCTTTAGTGGAACTTTCAGTTGTATCCAGTGAAGATAAATCTGCATCAACCGCA   1260
         S  D  P  L  V  E  L  S  V  V  S  S  E  D  K  S  A  S  T  A

1261    CAAAAACTTCTTTGCTATGGAACTGGCCTTAACCCAGAGATCAAGTGGCTCCCCGAATCT   1320
         Q  K  L  L  C  Y  G  T  G  L  N  P  E  I  K  W  L  P  E  S

1321    GTGGGAAATAATAAAAGTGAAGTAACAATGAATGAAGATGGACGTGTGAAGGTGTCCAGC   1380
         V  G  N  N  K  S  E  V  T  M  N  E  D  G  R  V  K  V  S  S

1381    GAGCTTTCAGTTACAGAACAAGAGTGGAACCGTGGAACTACATTCACCTGCCAAGTCAGT   1440
         E  L  S  V  T  E  Q  E  W  N  R  G  T  T  F  T  C  Q  V  S

                                                         ┌→Cm_ighδ4
1441    GATCAGGGTCGTCTCAACACTGTCCAGAAGAGCATCAGTTTTTGTGCAGTCACTCCAGAT   1500
         D  Q  G  R  L  N  T  V  Q  K  S  I  S  F  C  A  V  T  P  D

1501    CATGCTCGGAGTGCACAGGTTTACCTTTTGGGTCCCTCCATCAGTAATATGCCAGAGGAG   1560
         H  A  R  S  A  Q  V  Y  L  L  G  P  S  I  S  N  M  P  E  E

1561    GATCCTGTCTCTGTCACATGTCTGCTGTTGGGCCATAGGCTCCAGGACTTCTCAGTTAAT   1620
         D  P  V  S  V  T  C  L  L  L  G  H  R  L  Q  D  F  S  V  N

1621    TGCAAAGTAGGGACCGACAATTTGTCTTCAAATGTGATCAAAACTGAAATCCACGGCAAT   1680
         C  K  V  G  T  D  N  L  S  S  N  V  I  K  T  E  I  H  G  N

1681    GGAACAGAGAATGTTCAAAGGGTTATAAGGGTTCCAGCTGAAAAGTGGAAAAACCATGAA   1740
         G  T  E  N  V  Q  R  V  I  R  V  P  A  E  K  W  K  N  H  E

                                    ┌→Cm_ighμ2
1741    AATGTTTCCTGTGAGGTGAAACACCCCTGCGTTGTGCCCGACATCCCTGCAAAATCTCTG   1800
         N  V  S  C  E  V  K  H  P  C  V  V  P  D  I  P  A  K  S  L

1801    CTTCTAACAGCTCCGAGCCAAACAGAATTAGAAAATGGAACAGCTACCTTCATCTGCTTA   1860
         L  L  T  A  P  S  Q  T  E  L  E  N  G  T  A  T  F  I  C  L

1861    GCTTCAGAATTTTCACCTAAAACGTCCACATTTAAGTGGACCCGTGGAGGAACAAGCATC   1920
         A  S  E  F  S  P  K  T  S  T  F  K  W  T  R  G  G  T  S  I

1921    GATAATAAGGCCAAAGACCCCATTTTAATCGCGGGAAAACCTACTTACAGTTCCTTTAGT   1980
         D  N  K  A  K  D  P  I  L  I  A  G  K  P  T  Y  S  S  F  S

1981    ATTTTGGTACTCACTGCTGCCGAATGGACTGGCTCAACTTCTCCAGTTAAGTGCGAGTTC   2040
         I  L  V  L  T  A  A  E  W  T  G  S  T  S  P  V  K  C  E  F

                                               ┌→Cm_ighμ3
2041    CAGCAGAAGTCACAGACTCTGTCCAAGGAGGCAAGTTATCATTATGTGACCCGGGAGCAG   2100
         Q  Q  K  S  Q  T  L  S  K  E  A  S  Y  H  Y  V  T  R  E  Q

2101    CCAACAATTACAATAATCCCACCTTCCAACAATGACATGCTTATCAAAAGATCTGGCGAC   2160
         P  T  I  T  I  I  P  P  S  N  N  D  M  L  I  K  R  S  G  D

2161    CTCGTGTGCAAGGCTGAAGGACCATTGGGTTTCACTGGAATTAAGTGGGTTGCTAACGGT   2220
         L  V  C  K  A  E  G  P  L  G  F  T  G  I  K  W  V  A  N  G

2221    AAAGAGGTTGTTTCCCTATCTGAAAAAGTTGTGTCTACGAAGGCAGCGATCTCACTCACC   2280
         K  E  V  V  S  L  S  E  K  V  V  S  T  K  A  A  I  S  L  T

2281    ACCTCAATCTCTTACGAAGAGTGGCATAAGGGCATGAAATTTACCTGTGAGGTGCATCAT   2340
         T  S  I  S  Y  E  E  W  H  K  G  M  K  F  T  C  E  V  H  H

                                                        ┌→Cm_ighμtm1
2341    TCCTCATTTGCTCAGGGGTTTATAACAGAAGTCTACAAAAGAGAAAATTATGCCTTAACA   2400
         S  S  F  A  Q  G  F  I  T  E  V  Y  K  R  E  N  Y  A  L  T

                                                        ┌→Cm_ighμtm2
2401    TTTGTATTTCTCTTCCTCATCACCCTGTTCTACAGCATCGGGGTGACTGTTATCAAGGTG   2460
         F  V  F  L  F  L  I  T  L  F  Y  S  I  G  V  T  V  I  K  V

2461    AAATGGGAAACAACTAATGGAACGATTTTGATTTTCTCTCTGTTTTTTTTTTTCTTCAGTA   2520
         K  W  E  T  T  N  G  T  I  L  I  F  S  L  F  F  F  S  S  V

2521    TAAGTTTGTTTGTTTTGTTTTTTTTATTTTAGTGTTCTGAGTTACCCTTTCTGTATCATC   2580
         *
2581    TAGAAGATCTCTACATCACGGCAAATACTTTTGGACATTTAAATGTTGGACATTTTTTTC   2640
2641    ATGCATTTACTCAAATCTCAAATAAATGTCTTACAAAGTATGCAAATAAAAAAAAAAAAA   2700
2701    AAAAAAAAAAAAAAAAAAAAAAAA                                       2724
```

Supplementary Fig. 5. Nucleotide and translated amino acid sequences of the membrane-bound IgM constant domain of the bighead catfish (*Cm*_mIgM 3, GenBank accession number MN934746). The sequence was consisted of seven CH and two TM domains: Cμ1-Cδ2-Cδ3-Cδ4-Cδ5-Cμ2-Cμ3-TM1-TM2. The atypical polyadenylation signal (AATTAAA) in the 3′-UTR was bold.

```
        ▶Cm_ighμ1
   1  GCAACACAAGAAGCCCCGAAGTCCCTGTTTCCCGTGTGGCAGTGCGGCTCGTCCCCTGAT  60
       A  T  Q  E  A  P  K  S  L  F  P  V  W  Q  C  G  S  S  P  D

  61  GGTTTTGTCACTCTGGGCTGCGTCACCCGCGACTTGGCAACCCCTGATGGACTGAGCTAC  120
       G  F  V  T  L  G  C  V  T  R  D  L  A  T  P  D  G  L  S  Y

 121  GTTTGGAAGGATGCGAGCGGCACCGCGCTGACTACTAACGTTGTACAATACCCGGCGGTG  180
       V  W  K  D  A  S  G  T  A  L  T  T  N  V  V  Q  Y  P  A  V

 181  ATCGAGGGCGGAAAGTACTCCTCAGTGAGTCAGGCGCGCGTCAGTGCTAACGACTGGAAC  240
       I  E  G  G  K  Y  S  S  V  S  Q  A  R  V  S  A  N  D  W  N

 241  GCGAACAAGAAGTTCACTTGCGAAGTCACTAATCCCCGGGGAACGAAAACGGCAGAGCTG  300
       A  N  K  K  F  T  C  E  V  T  N  P  R  G  T  K  T  A  E  L
                  ▶Cm_ighμ2
 301  AAAAAGCCAGTTGTGCCCGACATCCCTGCAAAATCTCTGCTTCTAACAGCTCCGAGCCAA  360
       K  K  P  V  V  P  D  I  P  A  K  S  L  L  L  T  A  P  S  Q

 361  ACAGAATTAGAAAATGGAACAGCTACCTTCATCTGCTTAGCTTCAGAATTTTCACCTAAA  420
       T  E  L  E  N  G  T  A  T  F  I  C  L  A  S  E  F  S  P  K

 421  ACGTCCACATTTAAGTGGACCCGTGGAGGAACAAGCATCGATAATAAGGCCAAAGACCCC  480
       T  S  T  F  K  W  T  R  G  G  T  S  I  D  N  K  A  K  D  P

 481  ATTTTAATCGCGGGAAAACCTACTTACAGTTCCTTTAGTATTTTGGTACTCACTGCTGCC  540
       I  L  I  A  G  K  P  T  Y  S  S  F  S  I  L  V  L  T  A  A

 541  GAATGGACTGGCTCAACTTCTCCAGTTAAGTGCGAGTTCCAGCAGAAGTCACAGACTCTG  600
       E  W  T  G  S  T  S  P  V  K  C  E  F  Q  Q  K  S  Q  T  L
                        ▶Cm_ighμ3
 601  TCCAAGGAGGCAAGTTATCATTATGTGACCCGGGAGCAGCCAACAATTACAATAATCCCA  660
       S  K  E  A  S  Y  H  Y  V  T  R  E  Q  P  T  I  T  I  I  P

 661  CCTTCCAACAATGACATGCTTATCAAAAGATCTGGCGACCTCGTGTGCAAGGCTGAAGGA  720
       P  S  N  N  D  M  L  I  K  R  S  G  D  L  V  C  K  A  E  G

 721  CCATTGGGTTTCACTGGAATTAAGTGGGTTGCTAACGGTAAAGAGGTTGTTTCCCTATCT  780
       P  L  G  F  T  G  I  K  W  V  A  N  G  K  E  V  V  S  L  S

 781  GAAAAAGTTGTGTCTACGAAGGCAGCGATCTCACTCACCACCTCAATCTCTTACGAAGAG  840
       E  K  V  V  S  T  K  A  A  I  S  L  T  T  S  I  S  Y  E  E

 841  TGGCATAAGGGCATGAAATTTACCTGTGAGGTGCATCATTCCTCATTTGCTCAGGGGTTT  900
       W  H  K  G  M  K  F  T  C  E  V  H  H  S  S  F  A  Q  G  F
                               ▶Cm_ighμ4
 901  ATAACAGAAGTCTACAAAAGAGAAAATGGCCGAACGCCTAACTGCCCAGGGGTTTTCTTG  960
       I  T  E  V  Y  K  R  E  N  G  R  T  P  N  C  P  G  V  F  L

 961  CTCCCGCCACCAGAGAGCTCAAATGGGGATTTGAAGACCCTGACTTGCTATATAAAAAAC  1020
       L  P  P  P  E  S  S  N  G  D  L  K  T  L  T  C  Y  I  K  N

1021  TTTTACCCTAAGGAGGTTGCTGTGTCTTGGCTTATTGGTGATAAACAAGTGGACAATGAG  1080
       F  Y  P  K  E  V  A  V  S  W  L  I  G  D  K  Q  V  D  N  E

1081  AGCATCACTAAAGTTATTGAGAAAAATGGCAACTTTTCAGCATACAGTCAGCTGATTGTC  1140
       S  I  T  K  V  I  E  K  N  G  N  F  S  A  Y  S  Q  L  I  V

1141  AATCAGGACTCCTGGGGAAATGGCACAGAGTTCACCTGCAATGTTTATCATGAGTCCATC  1200
       N  Q  D  S  W  G  N  G  T  E  F  T  C  N  V  Y  H  E  S  I

1201  ATGGATAATCTTCGCCACCTTTCCAGAACCATTACTGGCACTTCAAATCCACCCTCCATA  1260
       M  D  N  L  R  H  L  S  R  T  I  T  G  T  S  N  P  P  S  I

1261  GTGAATCTCAGCCTAAATGTTCCCCAAAACTGCCCAAATCCTGGTCTGTAAGATGTAGAG  1320
       V  N  L  S  L  N  V  P  Q  N  C  P  N  P  G  L  *

1321  ATCCTGGTGCTGACCAACCTTTTCTACAATGAGACAGAACTTGATGATGACAACGTGGCA  1380
1381  ACCACTGCCTTAACATTTGTATTTCTCTTCCTCATCACCCTGTTCTACAGCATCGGGGTG  1440
1441  ACTGTTATCAAGGTGAAATGAGAAACAACTAATGGAACGATTTTGATTTTCTCTCTGTTT  1500
1501  TTTTTTTCTTCAGTATAATGTTCTGAGTTACCCTTTCTGTTAAACTATCTTCTCTTAGCT  1560
1561  ACTTTCCATGTATTTTATATCTGTAAATACTTTTTTGTCATTTAAATGTTGGATTTTTTT  1620
1621  TTCATGCTTTTACTCAAATCTCAAATAAATGTCAAACATAGTAAAACATTTATCAGATAA  1680
1681  TGTAAAAAAAAAAAAAAAAAAAAAAAAAAAAAAAA                           1714
```

Supplementary Fig. 6. Nucleotide and translated amino acid sequences of the secreted IgM constant domain of the bighead catfish (*Cm_s*IgM 1, GenBank accession number MN934747). The sequence was consisted of three CH and two TM domains: Cμ1-Cμ2-Cμ3-Cμ4. The atypical polyadenylation signal (AATTAAA) in the 3′-UTR was bold.

```
       Cg_ighμ1
1     GCAACGCAAGAAGCCCCGAAGTCTCTGTTTCCCGTGTGGCAGTGCGGCTCGTCCCCTGAT   60
       A  T  Q  E  A  P  K  S  L  F  P  V  W  Q  C  G  S  S  P  D

61    GGTTTCGTCACTCTGGGCTGCCTCACCCGCGACTTGGCAACCGGCGATGGACTGAGCTAC   120
       G  F  V  T  L  G  C  L  T  R  D  L  A  T  G  D  G  L  S  Y

121   GTTTGGAAGGATGCGAGCGGCACCGCGCTGACTACCGTTGTACAATACCCGGCAGTGCTG   180
       V  W  K  D  A  S  G  T  A  L  T  T  V  V  Q  Y  P  A  V  L

181   GACAACGGAAAGTACTCCTCAGTGAGTCAGGCGCGCGTCACTGCTACCGAGTGGGACGCG   240
       D  N  G  K  Y  S  S  V  S  Q  A  R  V  T  A  T  E  W  D  A

241   AACAAGCCGTTCACTTGCCAAGTCACTAATTCCATTGGAACCGCAACGGCGAACGTGCAA   300
       N  K  P  F  T  C  Q  V  T  N  S  I  G  T  A  T  A  N  V  Q

                  Cg_ighμ2
301   AAACCACCTCTCGTCATCCCAGCAAAATCTCTGCTTCTAACAGCTCCGAGCCAAACAGAA   360
       K  P  P  L  V  I  P  A  K  S  L  L  L  T  A  P  S  Q  T  E

361   ATAGACAATGGAACAGCTACCTTCATCTGCTTAGCTTCAGAATTTTCACCTAAAACGCAC   420
       I  D  N  G  T  A  T  F  I  C  L  A  S  E  F  S  P  K  T  H

421   ACATTTAAGTGGACCCGTGAAAAGACAAGCCTCGATAGTAAGGCAAAAGCCCCAATTTTA   480
       T  F  K  W  T  R  E  K  T  S  L  D  S  K  A  K  A  P  I  L

481   ATCCCGGGACAAAAAATTTACAGTGCCTTAAGCATTTTGGAACTCACTGCCAGCGAATGG   540
       I  P  G  Q  K  I  Y  S  A  L  S  I  L  E  L  T  A  S  E  W

541   ATGGGCTCAACTTCTCCAGTTAAGTGTGAGTTCCAGCACAAGGAACAGACTCTGTCCCAG   600
       M  G  S  T  S  P  V  K  C  E  F  Q  H  K  E  Q  T  L  S  Q

                           Cg_ighμ3
601   GAAGCAAGTCATGGGACATGTGAGGAGCAGCCAAAAATGACAATAAGTCCACCTTCCAAT   660
       E  A  S  H  G  T  C  E  E  Q  P  K  M  T  I  S  P  P  S  N

661   GGCATTCTTATCAATAGATCTGCTGATATTGTGTGCAAGGCTGAAGGACCAATGGGTTTC   720
       G  I  L  I  N  R  S  A  D  I  V  C  K  A  E  G  P  M  G  F

721   ACCGCCATTAAATGGGTTGTTAACGGTAAAGAGGTTGCTTCCCTACCTCAAAGCGATGTG   780
       T  A  I  K  W  V  V  N  G  K  E  V  A  S  L  P  Q  S  D  V

781   TCTTCGAAGACAGCGATCTCACTCACCACCACAATCTCTTATGAAGAGTGGCATACTGGC   840
       S  S  K  T  A  I  S  L  T  T  T  I  S  Y  E  E  W  H  T  G

841   ACCAAATTTACCTGTGAGGTGTATCATTCAGCATTAGCTCAGGGATTTATACAAGAAGAC   900
       T  K  F  T  C  E  V  Y  H  S  A  L  A  Q  G  F  I  Q  E  D

                        Cg_ighμtm1
901   TACCAAAGAGTAAACTATGCCTTAACATTTGTATTTCTCTTCCTCATCACTCTGTTCTAC   960
       Y  Q  R  V  N  Y  A  L  T  F  V  F  L  F  L  I  T  L  F  Y

                     Cg_ighμtm2
961   AGCATCGGGGTGGTGAAATGGGAAACAACTAATGGAACGATTTTGATTTTCTCTCTGTTT   1020
       S  I  G  V  V  K  W  E  T  T  N  G  T  I  L  I  F  S  L  F

1021  TTTTTTTCTTCTTCAGTATAAAGCTGATGCCGGGTTACTACATTGAGACAGAAACTGATG   1080
       F  F  S  S  S  V
1081  ATGAAAACGTGGCAAACACTGCCTTAACATTTGTATTTCTCTTCCTCATCACTCTGTTCT   1140
1141  ACAGCATCGGGGTGACTGTTATCAAGGTGAAATAATAAACATTAAAAGCCAAAAAAAAAA   1200
1201  AAAAAAAAAAAAAAAAAAA                                            1219
```

Supplementary Fig. 7. Nucleotide and translated amino acid sequences of the membrane-bound IgM constant domain of the north African catfish (*Cg*_mIgM 1.1, GenBank accession number MN934748). The sequence was consisted of three CH and two TM domains: Cμ1-Cμ2-Cμ3-TM1-TM2. The atypical polyadenylation signal (AATTAAA) in the 3′-UTR was bold.

```
        ┌─►Cg_ighμ1
1       GCAACGCAAGAAGCCCCGAAGTCTCTGTTTCCCGTGTGGCAGTGCGGCTCGTCCCCTGAT   60
         A  T  Q  E  A  P  K  S  L  F  P  V  W  Q  C  G  S  S  P  D

61      GGTTTCGTCACTCTGGGCTGCCTCACCCGCGACTTGGCAACCGGCGATGGACTGAGCTAC   120
         G  F  V  T  L  G  C  L  T  R  D  L  A  T  G  D  G  L  S  Y

121     GTTTGGAAGGATGCGAGCGGCACCGCGCTGACTACCGTTGTACAATACCCGGCAGTGCTG   180
         V  W  K  D  A  S  G  T  A  L  T  T  V  V  Q  Y  P  A  V  L

181     GACAACGGAAAGTACTCCTCAGTGAGTCAGGCGCGCGTCACTGCTACCGAGTGGGACGCG   240
         D  N  G  K  Y  S  S  V  S  Q  A  R  V  T  A  T  E  W  D  A

241     AACAAGCCGTTCACTTGCCAAGTCACTAATTCCATTGGAACCGCAACGGCGAACGTGCAA   300
         N  K  P  F  T  C  Q  V  T  N  S  I  G  T  A  T  A  N  V  Q
                  ┌─►Cg_ighμ2
301     AAACCACCTCTCGTCATCCCAGCAAAATCTCTGCTTCTAACAGCTCCGAGCCAAACAGAA   360
         K  P  P  L  V  I  P  A  K  S  L  L  L  T  A  P  S  Q  T  E

361     ATAGACAATGGAACAGCTACCTTCATCTGCTTAGCTTCAGAATTTTCACCTAAAACGCAC   420
         I  D  N  G  T  A  T  F  I  C  L  A  S  E  F  S  P  K  T  H

421     ACATTTAAGTGGACCCGTGAAAAGACAAGCCTCGATAGTAAGGCAAAAGCCCCAATTTTA   480
         T  F  K  W  T  R  E  K  T  S  L  D  S  K  A  K  A  P  I  L

481     ATCCCGGGACAAAAAATTTACAGTGCCTTAAGCATTTTGGAACTCACTGCCAGCGAATGG   540
         I  P  G  Q  K  I  Y  S  A  L  S  I  L  E  L  T  A  S  E  W

541     ATGGGCTCAACTTCTCCAGTTAAGTGTGAGTTCCAGCACAAGGAACAGACTCTGTCCCAG   600
         M  G  S  T  S  P  V  K  C  E  F  Q  H  K  E  Q  T  L  S  Q
                          ┌─►Cg_ighμ3
601     GAAGCAAGTCATGGGACATGTGAGGAGCAGCCAAAAATGACAATAAGTCCACCTTCCAAT   660
         E  A  S  H  G  T  C  E  E  Q  P  K  M  T  I  S  P  P  S  N

661     GGCATTCTTATCAATAGATCTGCTGATATTGTGTGCAAGGCTGAAGGACCAATGGGTTTC   720
         G  I  L  I  N  R  S  A  D  I  V  C  K  A  E  G  P  M  G  F

721     ACCGCCATTAAATGGGTTGTTAACGGTAAAGAGGTTGCTTCCCTACCTCAAAGCGATGTG   780
         T  A  I  K  W  V  V  N  G  K  E  V  A  S  L  P  Q  S  D  V

781     TCTTCGAAGACAGCGATCTCACTCACCACCACAATCTCTTATGAAGAGTGGCATACTGGC   840
         S  S  K  T  A  I  S  L  T  T  T  I  S  Y  E  E  W  H  T  G

841     ACCAAATTTACCTGTGAGGTGTATCATTCAGCATTAGCTCAGGGATTTATACAAGAAGAC   900
         T  K  F  T  C  E  V  Y  H  S  A  L  A  Q  G  F  I  Q  E  D
                       ┌─►Cg_ighμtm1
901     TACCAAAGAGTAAACTATGCCTTAACATTTGTATTTCTCTTCCTCATCACTCTGTTCTAC   960
         Y  Q  R  V  N  Y  A  L  T  F  V  F  L  F  L  I  T  L  F  Y
                   ┌─►Cg_ighμtm2
961     AGCATCGGGGTGGTGAAATGGGAAACAACTAATGGAACGATTTTGATTTTCTCTCTGTTT   1020
         S  I  G  V  V  K  W  E  T  T  N  G  T  I  L  I  F  S  L  F

1021    TTTTTTTCTTCTTCAGTATAAAGCTGATGCCGGGTTACTACATTGAGACAGAAACTGATG   1080
         F  F  S  S  S  V  *
1081    ATGAAAACGTGGCAAACACTGCCTTAACATTTGTATTTCTCTTCCTCATCACTCTGTTCT   1140
1141    ACAGCATCGGGGTGACTGTTATCAAGGTGAAATAATAAACATAAACAACTGATGGAATGA   1200
1201    GTTCGATCTTCTCTCTGTTTCGCCAGTATAATGTTCTGAGTTAAACTTTCTGTTAAACTA   1260
1261    TCTTGTCTCAGCTACTTTTCATGTGTTTTATATCTGTAAATACTTTTTGTCATTTAAATG   1320
1321    TTGATTTCTTTTCATGCTTTTACTCAAATCTCAAATAAATGCAATTAAAAGCCAAAAAAA   1380
1381    AAAAAAAAAAAAAAAAAAAAAA                                         1402
```

Supplementary Fig. 8. Nucleotide and translated amino acid sequences of the membrane-bound IgM constant domain of the north African catfish (*Cg*_mIgM 1.2, GenBank accession number MN934749). The sequence was consisted of three CH and two TM domains: Cμ1-Cμ2-Cμ3-TM1-TM2. The atypical polyadenylation signal (AATTAAA) in the 3′-UTR was bold.

```
          ┌─►Cg_ighµ1
   1      GCAACGCAAGAAGCCCCGAAGTCTCTGTTTCCCGTGTGGCAGTGCGGCTCGTCCCCTGAT   60
           A  T  Q  E  A  P  K  S  L  F  P  V  W  Q  C  G  S  S  P  D

  61      GGTTTCGTCACTCTGGGCTGCCTCACCCGCGACTTGGCAACCGGCGATGGACTGAGCTAC   120
           G  F  V  T  L  G  C  L  T  R  D  L  A  T  G  D  G  L  S  Y

 121      GTTTGGAAGGATGCGAGCGGCACCGCGCTGACTACCGTTGTACAATACCCGGCAGTGCTG   180
           V  W  K  D  A  S  G  T  A  L  T  T  V  V  Q  Y  P  A  V  L

 181      GACAACGGAAAGTACTCCTCAGTGAGTCAGGCGCGCGTCACTGCTACCGAGTGGGACGCG   240
           D  N  G  K  Y  S  S  V  S  Q  A  R  V  T  A  T  E  W  D  A

 241      AACAAGCCGTTCACTTGCCAAGTCACTAATTCCATTGGAACCGCAACGGCGAACGTGCAA   300
           N  K  P  F  T  C  Q  V  T  N  S  I  G  T  A  T  A  N  V  Q
                   ┌─►Cg_ighµ2
 301      AAACCACCTCTCGTCATCCCAGCAAAATCTCTGCTTCTAACAGCTCCGAGCCAAACAGAA   360
           K  P  P  L  V  I  P  A  K  S  L  L  L  T  A  P  S  Q  T  E

 361      ATAGACAATGGAACAGCTACCTTCATCTGCTTAGCTTCAGAATTTTCACCTAAAACGCAC   420
           I  D  N  G  T  A  T  F  I  C  L  A  S  E  F  S  P  K  T  H

 421      ACATTTAAGTGGACCCGTGAAAAGACAAGCCTCGATAGTAAGGCAAAAGCCCCAATTTTA   480
           T  F  K  W  T  R  E  K  T  S  L  D  S  K  A  K  A  P  I  L

 481      ATCCCGGGACAAAAAATTTACAGTGCCTTAAGCATTTTGGAACTCACTGCCAGCGAATGG   540
           I  P  G  Q  K  I  Y  S  A  L  S  I  L  E  L  T  A  S  E  W

 541      ATGGGCTCAACTTCTCCAGTTAAGTGTGAGTTCCAGCACAAGGAACAGACTCTGTCCCAG   600
           M  G  S  T  S  P  V  K  C  E  F  Q  H  K  E  Q  T  L  S  Q
                           ┌─►Cg_ighµ3
 601      GAAGCAAGTCATGGGACATGTGAGGAGCAGCCAAAAATGACAATAAGTCCACCTTCCAAT   660
           E  A  S  H  G  T  C  E  E  Q  P  K  M  T  I  S  P  P  S  N

 661      GGCATTCTTATCAATAGATCTGCTGATATTGTGTGCAAGGCTGAAGGACCAATGGGTTTC   720
           G  I  L  I  N  R  S  A  D  I  V  C  K  A  E  G  P  M  G  F

 721      ACCGCCATTAAATGGGTTGTTAACGGTAAAGAGGTTGCTTCCCTACCTCAAAGCGATGTG   780
           T  A  I  K  W  V  V  N  G  K  E  V  A  S  L  P  Q  S  D  V

 781      TCTTCGAAGACAGCGATCTCACTCACCACCACAATCTCTTATGAAGAGTGGCATACTGGC   840
           S  S  K  T  A  I  S  L  T  T  T  I  S  Y  E  E  W  H  T  G

 841      ACCAAATTTACCTGTGAGGTGTATCATTCAGCATTAGCTCAGGGATTTATACAAGAAGAC   900
           T  K  F  T  C  E  V  Y  H  S  A  L  A  Q  G  F  I  Q  E  D
                        ┌─►Cg_ighµ4
 901      TACCAAAGAGTAAACGGCCAAACGCAATGCCCAGAGCTTTTCTTGCTCCCTCCACCAGAG   960
           Y  Q  R  V  N  G  Q  T  Q  C  P  E  L  F  L  L  P  P  P  E

 961      AGCTCAAAGGGGGATTCAATGACCCTGACTTGCTATATTAAGGACTTCTACCCTAAGGAG   1020
           S  S  K  G  D  S  M  T  L  T  C  Y  I  K  D  F  Y  P  K  E

1021      GTTGCTGTGTCTTGGCTTGTTGGTGATAAACTAGTGGACAATGAGAGCACTGTTAAAGTT   1080
           V  A  V  S  W  L  V  G  D  K  L  V  D  N  E  S  T  V  K  V

1081      ATTGAGAAAAATGGCATCTTTTCAGCATACAGTCAGCTAATTGTCGATAGGGCAGACTGG   1140
           I  E  K  N  G  I  F  S  A  Y  S  Q  L  I  V  D  R  A  D  W

1141      GAAAATGGCACTGTGTTCACCTGCAATGTTTATCATGAGTCCATCGTAGAGAGTGTGCGC   1200
           E  N  G  T  V  F  T  C  N  V  Y  H  E  S  I  V  E  S  V  R

1201      CACCTTTCTAGATCCGTTGCTGGTAATTCAAATCCACCCTCCGTAGTGAATCTCAGCTTA   1260
           H  L  S  R  S  V  A  G  N  S  N  P  P  S  V  V  N  L  S  L

1261      AATGTTCCCCAAAACTGCCCAAATGCTCTGTAAAGCTGATGCCGGGTTACTACATTGAGA   1320
           N  V  P  Q  N  C  P  N  A  L  *

1321      CAGAAACTGATGATGAAAACGTGGCAAACACTGCCTTAACATTTGTATTTCTCTTCCTCA   1380
1381      TCACTCTGTTCTACAGCATCGGGGTGACTGTTATCAAGGTGAAATAATAAATGCAATTAA   1440
1441      AAGCCAAAAAAAAAAAAAAAAAAAAAAAAAAAAA                                     1474
```

Supplementary Fig. 9. Nucleotide and translated amino acid sequences of the secreted IgM constant domain of the north African catfish (*Cm*_sIgM 1.1, GenBank accession number MN934750) and hybrid catfish (hybrid:*Cg*_sIgM 1.1, GenBank accession number MN934752). The sequence was consisted of four CH domains: Cμ1-Cμ2-Cμ3-Cμ4. The atypical polyadenylation signal (AATTAAA) in the 3′-UTR was bold.

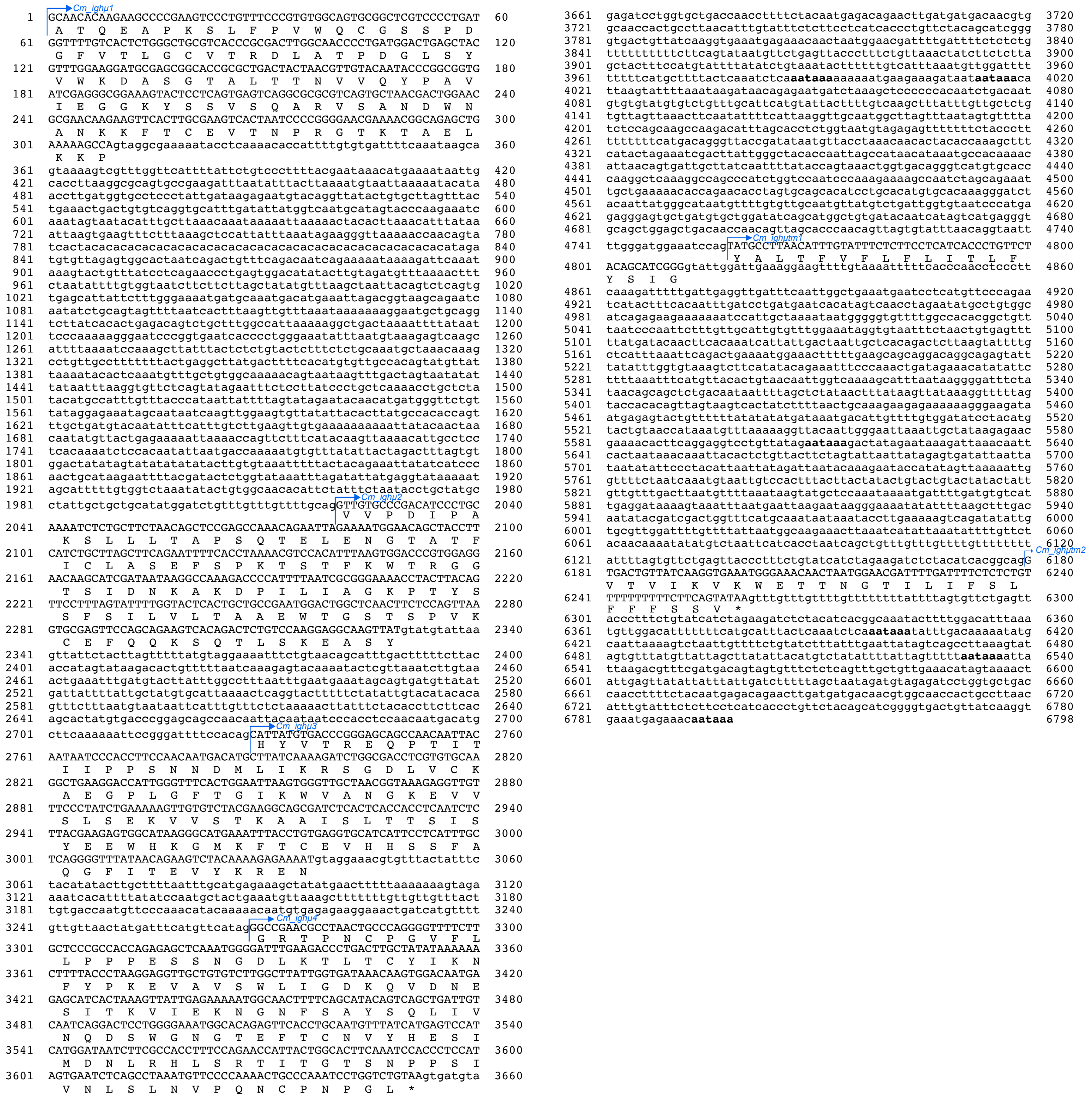

Supplementary Fig. 10. Complete nucleotide and translated amino acid sequences of the *ighμ* gene of the bighead catfish and their hybrid catfish (GenBank accession number MZ559374 and MZ559376, respectively). The atypical polyadenylation signals (AATTAAA) in the 3′-UTR were bold.

Cg_ighμ1
1 GCAACGCAAGAAGCCCCGAAGTCTCTGTTTCCCGTGTGGCAGTGCGGCTCGTCCCCTGAT 60
A T Q E A P K S L F P V W Q C G S S P D
61 GGTTTCGTCACTCTGGGCTGCCTCACCCGCGACTTGGCAACCGGCGATGGACTGAGCTAC 120
G F V T L G C L T R D L A T G D G L S Y
121 GTTTGGAAGGATGCGAGCGGCACCGCGCTGACTACCGTTGTACAATACCCGGCAGTGCTG 180
V W K D A S G T A L T T V V Q Y P A V L
181 GACAACGGAAAGTACTCCTCAGTGAGTCAGGCGCGCGTCACTGCTACCGAGTGGGACGCG 240
D N G K Y S S V S Q A R V T A T E W D A
241 AACAAGCCGTTCACTTGCCAAGTCACTAATTCCATTGGAACCGCAACGGCGAACGTGCAA 300
N K P F T C Q V T N S I G T A T A N V Q
301 AAACCACCTgtgaaaaatactacaaaacataattttgtgtgtcttttccaaataagcagt 360
K P P
361 gaaagtcgtgtattattatatgtaaatattgacatatactcacacacgaacacacagaca 420
421 ctctcagggaagaaaaatgttaccaaacttttcttgccccttggtgaggccaagcatttg 480
481 attcattttattctgtaccttttacgaggaaacatgaaaataattgcaccttaaggccca 540
541 gtgccgtatatttaaatatttactctaaaagccaattaaaaatatgtaactttaaaacga 600
601 agcaatcgatgcatcttcaaataacaagagaatgtacagtttagactgtgcctagattac 660
661 tgatacagactgtggcaggtgcatttgatgcaatggtcaattaatttattatagattatc 720
721 catagtaaacaagcaattaaatagtaatacatttgcttcaactaacaaaaattaaacatt 780
781 acactttaccattcatgatacttttaactttaattgtacaatatctgaatgttttattca 840
841 acacagtttaagcttacattaagtacattttctgtaaaggtacttcatttaaaaaagaag 900
901 agttaaaaccaacagtaagtaatgtatcatgatagatagggggtggtagagtggcactaat 960
961 cagacggtttcagagaatcagagaaatcaaaaattcaaatacaacagtgtttttccttaa 1020
1021 agacctgagtggacataaactttaccaaatatttggtggtaatctctttcttagctacat 1080
1081 gtttaagcctattagcttatcagtctctgtgtgagcataattttttttgggaaaatgatgc 1140
1141 aaatgacatgaaatcagacagtaagcaaaattcaatgtctgccattagtttttacttctt 1200
1201 ttaagtccctttaaaaaaaaaaaaaaaaaaagaatgctgcatgtctaatcacactgagacca 1260
1261 gtctgctttggccattaaaatgtctgactaaaagttgattattcctaaaaaggctatcca 1320
1321 gttgaatcagcactggaatattttatgtcaagcattttaaaatccaaagtttatttactc 1380
1381 tctgtactctttctctgccaatgctgaacaaagcgtgtttctttttttactgaggcttat 1440
1441 gacttttttgcatgtgttgccacagtatgttatcagaatacactcaaatgtttgctgtggc 1500
1501 aaaaacagtaataagttagattttacagactagttatttaacttgttcccagtatagaat 1560
1561 ttttactttatccatgctcaaaacctactctatacttgacatttgtttacccataattat 1620
1621 tttattatagaatacaacatgatgggttctgttatagaaaaaatagctataatcaagttg 1680
1681 ttgaataatgtgaaatgttttattataattatgccacatcagtttgctgatataaaatat 1740
1741 taaatttgtcttgaagttaaaaaatgtaaatatatacaactaccaatattttactgagaa 1800
1801 gatttaaaaccagttctttcgtacaagttaaaatattgcctcctcacaaaaaaaaacaaa 1860
1861 aaaaaaactctgcaatattaatgcctttggaatttagtttgataaataactacacattta 1920
1921 aaaaatgtgtttgtattactagatttatattatgtatacaggtacttgtgtaaattgtta 1980
1981 aaatagaaataatatcatatcaactgcataagaattttatggcactctgatataaattca 2040
2041 gatataataagttataaaaaggcatttttgtggtcttaatatactgtggcaacacatttt 2100
2101 atttctaaactgcagtacctgctgtgcctattgctgctgcgatatggatctgtgtgtttg 2160
Cg_ighμ2
2161 ttttgcaagctgagcagCTCGTCATCCCAGCAAAATCTCTGCTTCTAACAGCTCCGAGCC 2220
L V I P A K S L L L T A P S
2221 AAACAGAAATAGACAATGGAACAGCTACCTTCATCTGCTTAGCTTCAGAATTTTCACCTA 2280
Q T E I D N G T A T F I C L A S E F S P
2281 AAACGCACACATTTAAGTGGACCCGTGAAAAGACAAGCCTCGATAGTAAGGCAAAAGCCC 2340
K T H T F K W T R E K T S L D S K A K A
2341 CAATTTTAATCCCGGGACAAAAAATTTACAGTGCCTTAAGCATTTTGGAACTCACTGCCA 2400
P I L I P G Q K I Y S A L S I L E L T A
2401 GCGAATGGATGGGCTCAACTTCTCCAGTTAAGTGTGAGTTCCAGCACAAGGAACAGACTC 2460
S E W M G S T S P V K C E F Q H K E Q T
2461 TGTCCCAGGAAGCAAGTCATGGGACAgtatgtattaagttattctacttaggttttatga 2520
L S Q E A S H G T
2521 ctatgattatttttttacaaatgaaaatttctttaacagcatttgacttcagtgttattc 2580
2581 ttacaccatagtataagttactgtttttaatgaaacaatacaaagtaattgttaaatctt 2640
2641 gtactcaaattaaatgtaataatttgttctttcatttgaatgaaatggtaatgatgtatt 2700
2701 attattttactgtaatgtgcaataaaattcaggtatttttccagattgtaaattcacagt 2760
2761 ttctttaattacacaaaatatttgtttcttgataaacttatttctacaccttctttacag 2820
Cg_ighμ3
2821 TGTGAGGAGCAGCCAAAAATGACAATAAGTCCACCTTCCAATGGCATTCTTATCAATAGA 2880
C E E Q P K M T I S P P S N G I L I N R
2881 TCTGCTGATATTGTGTGCAAGGCTGAAGGACCAATGGGTTTCACCGCCATTAAATGGGTT 2940
S A D I V C K A E G P M G F T A I K W V
2941 GTTAACGGTAAAGAGGTTGCTTCCCTACCTCAAAGCGATGTGTCTTCGAAGACAGCGATC 3000
V N G K E V A S L P Q S D V S S K T A I
3001 TCACTCACCACCACAATCTCTTATGAAGAGTGGCATACTGGCACCAAATTTACCTGTGAG 3060
S L T T T I S Y E E W H T G T K F T C E
3061 GTGTATCATTCAGCATTAGCTCAGGGATTTATACAAGAAGACTACCAAAGAGTAAACgta 3120
V Y H S A L A Q G F I Q E D Y Q R V N
3121 ggaatcgtgtttacttttctacatattcttgcttttaatttgcatgagaaagcttaaaa 3180
3181 aaaagcagaaaatcacattttatatccagtgctactgaaattatatggaattattatat 3240
3241 gttaaagctttttttgttgttgtttacttgtgaccaatgttcccaaacgtacaaaaacaa 3300
Cg_ighμ4
3301 tttaagagaaagaaactgatcatgctttgttgttaactatgattttatgttcatagGGCC 3360
G
3361 AAACGCAATGCCCAGAGCTTTTCTTGCTCCCTCCACCAGAGAGCTCAAAGGGGGATTCAA 3420
Q T Q C P E L F L L P P P E S S K G D S
3421 TGACCCTGACTTGCTATATTAAGGACTTCTACCCTAAGGAGGTTGCTGTGTCTTGGCTTG 3480
M T L T C Y I K D F Y P K E V A V S W L
3481 TTGGTGATAAACTAGTGGACAATGAGAGCACTGTTAAAGTTATTGAGAAAAATGGCATCT 3540
V G D K L V D N E S T V K V I E K N G I
3541 TTTCAGCATACAGTCAGCTAATTGTCGATAGGGCAGACTGGGAAAATGGCACTGTGTTCA 3600
F S A Y S Q L I V D R A D W E N G T V F
3601 CCTGCAATGTTTATCATGAGTCCATCGTAGAGAGTGTGCGCCACCTTTCTAGATCCGTTG 3660
T C N V Y H E S I V E S V R H L S R S V
3661 CTGGTAATTCAAATCCACCCTCCGTAGTGAATCTCAGCTTAAATGTTCCCCAAAACTGCC 3720
A G N S N P P S V V N L S L N V P Q N C
3721 CAAATGCTCTGTAAgtagctgatgccgggttactacattgagacagaaactgatgatgaa 3780
P N A L *
3781 aacgtggcaaacactgccttaacatttgtatttctcttcctcatcactctgttctacagc 3840
3841 atcggggtgactgttatcaaggtgaaat**aataaa**cataaacaactgatggaatgagttcg 3900
3901 atcttctctctgtttcgccagtataatgttctgagttaaactttctgttaaactatcttg 3960
3961 tctcagctacttttcatgtgttttatatctgtaaatacttttttgtcatttaaatgttgat 4020
4021 ttcttttcatgcttttactcaaatctca**aataaa**taaaaaaatgtaagaaagacaaacct 4080
4081 tggtataaaacattaagtatttacatatgataacagagaatgctctaaagcttcccccca 4140
4141 caatctgacaatgtgtgtatgtgtctgtttgcattcctgtaatattttttgtcaagcttta 4200
4201 tttgttgctctgtgttagttacatttcaatattttcattaaggttgcaatgtcttagttt 4260
4261 aatactgttttatctcaagcaagccaagacattcagcacctctggtaatgtagagaattt 4320
4321 tttctaccttcttccatgaaaggttgagggttatcaatatcatgtgtacctaagtgacac 4380
4381 caaagctttcatactagaaactgacctattgagctacaccaattagtcataacattaacg 4440
4441 ccacaaaacattaccacattaacaatgattggttattaatttttaccagtaaactggtga 4500
4501 cagggtcatgtgcacccacggctcaaaggccagctcatctggtccaatcccacagaaaag 4560
4561 ccaatgtagcacaaattgctgaaaaaagtaaatgctggccatgatagacccccgaacacc 4620
4621 cagtgcagcacatcctgtacatggcgctctgcggagcctaaggctactgacccagcctct 4680
4681 agacttcccaggtctcaatagaaccaagcatccaaggaatgccccagacaaacaggccca 4740
4741 atccatcaaggccctaccccacaaaccacagcacacaaatgaatctggtgggtcccccat 4800
4801 tccaggtatcacatcacaaaactccgaaggtcatgggcctgaaggggccaaaacttccca 4860
4861 tgtggtcttaaaagggatcttcaccaattatggggcaaaaaggtttttttggttttaaagt 4920
4921 tattggccggaaaggggggggaatccccctttaaaaggggttgattcccgggtttttccc 4980
Cg_ighμtm1
4981 ttggggccgcagTATGCCTTAACATTTGTATTTCTCTTCCTCATCACTCTGTTCTACAGC 5040
Y A L T F V F L F L I T L F Y S
5041 ATCGGGgtaagtctgttatcaaggtgagaactgatcccagtaaacataaaccgggtgaat 5100
I G
5101 tcaggaacaaaaaaatgtggtgtgcaaacacagatgtttagttccaattctgtgaggtct 5160
5161 tgttgcattgtgtatgggattgctcttacatttaacattaaacatagctgtaatttctaa 5220
5221 cagtaattttttttaatgaaaaaacttcaccaatttattaattgacttcacttattgataa 5280
5281 ataattcacagacttaattatttgcttattttaatttagacagaaaattaaaactttttt 5340
5341 gagatggcagggcaggccaattccatgttccaaaatcactataagaaatggtaaaatcca 5400
5401 attgggtaaagggatttaggaagaggatttctataacagcagctctgacaataatctttg 5460
5461 ctatatagctctatcaattagaaaggtttttagtattacatatttagtaagtcactaatt 5520
5521 ttcaacttcaaagcagagaaaagggggggtgatgacaggagtcctgtttatgtttgtgttt 5580
5581 ttattatatatgacatctgtagatgtggatattgtaaccataaatgtttaaaaaggttac 5640
5641 agttggaaatcctttgctaattgctatactacaatataaataaaacatttcaggaggtgc 5700
5701 cgttatag**aataaa**tatagaataaaaatatatagaatacataaatcacttataaacataa 5760
5761 aatcttaagttttctaaactttgttagttatactgttcattatagaattatataaatta 5820
5821 taatattttctctacattaattatagaccaatacaaagaacaccatatatttaaagattg 5880
5881 catttttaatcaaatgtaattgtcaattttacttattgtactgttcataaaatgttgctt 5940
5941 gacccaatgttttaaataagtatgctgcaaataaaataattttaatttgtagttggggat 6000
6001 aaaagtaaatgtttttaataaaaaataaaaaacaaggaaaatatattttaagcttaacag 6060
6061 aatataatagacaggtttcatgcacatgtacagtaaatagtaaatacattggaaaagtca 6120
6121 gaaatgtattgtgtgttttgtattttgtttatttaatggcaggaaaactctgacaatgtg 6180
Cg_ighμtm2
6181 taaattcaaaaactgtcccaatatatttgaattcaccataaaaaagagagattagGTGGT 6240
V V
6241 GAAATGGGAAACAACTAATGGAACGATTTTGATTTTCTCTCTGTTTTTTTTTTCTTCTTC 6300
K W E T T N G T I L I F S L F F F S S S
6301 AGTATAAgtagctgatgccgggttactacattgagacagaaactgatgatgaaaacgtgg 6360
V *
6361 caaacactgccttaacatttgtatttctcttcctcatcactctgttctacagcatcgggg 6420
6421 tgactgttatcaaggtgaaat**aataaa**cataaacaactgatggaatgagttcgatcttct 6480
6481 ctctgtttcgccagtataatgttctgagttaaactttctgttaaactatcttgtctcagc 6540
6541 tacttttcatgtgttttatatctgtaaatacttttttgtcatttaaatgttgatttctttt 6600
6601 catgcttttactcaaatctca**aataaa**tgc**aataaa**agcc 6641

Supplementary Fig. 11. Complete nucleotide and translated amino acid sequences of the *ighμ* gene of the bighead catfish and their hybrid catfish (GenBank accession number MZ559375 and MZ559377, respectively). The atypical polyadenylation signals (AATTAAA) in the 3′-UTR were bold.

VH Number	VH		DH	JH			
VH 51	GCA	AAA	GAGGTTTACGGT	TAC	TTT	GAC	TAC
VH 33	GCA	AGA	TCTCGGGAATTGGGT	GCT	TTT	GAC	TAC
VH 6	GCA	AGA	TACGGGGAATTGGGT	GCT	TTT	GAC	TAC
VH 92	GCT	CGG	GGCTACGGT	GCT	TTT	GAC	TAC
VH 94	GCC	AGA	GAAGTGGGGGGCTTCGGT	GCT	TTT	GAC	TAC
VH 50	GCA	AGT	ACGGCCCCCGGT	GCT	TTT	GAC	TAC
VH 48	GCT	AGA	GAGGGATACCGGGGTGGGGGT	GCT	TTT	GAC	TAC
VH 75	GCG	CGC	TGGAGCAGCTCCCTTTACTAC	GGC	TTT	GAC	TAC
VH 29	GCA	AGA	AGCAGCTGGGCC	GGC	TTT	GAC	TAC
VH 100	GCA	CGT	CGGATAGCAGCTGGGATCTAC	GGC	TTT	GAC	TAC
VH 65	GCG	CGA	CAGTCAGATAGCAGCTGGGGTCCC	GGC	TTT	GAC	TAC
VH 79	GCT	CGT	CGCATGAGGGATAGCAGCTGG	TAC	TTT	GAC	TAC
VH 45	GCA	AAG	TTGGATAGCAGCTCG	TCC	TTT	GAC	TAC
VH 10	GCA	AGT	TACATCCCTGGGGGGGTATAC	GGC	TTT	GAC	TAC
VH 27	GCA	AGA	CACGGTGGGGGG	GGC	TTT	GAC	TAC
VH 49	GCA	AAA	GGTGGGGTGGGGGGG	TAC	TTT	GAC	TAC
VH 61	GCA	AGA	TGGGGGGGTACC	TAC	TTC	GAC	TAC
VH 9	GCA	AGA	TATCCGGGATTACC	TAC	TTC	GAC	TAC
VH 81	GCC	CGT	AAAAATAACTACGGCGCCGATAATACC	TAC	TTC	GAC	TAC
VH 63	GCA	CGA	GACGGGAACTAC	TAC	TTT	GAC	AAC
VH 46	GCA	AAG	TACATAGGGTCTGGAGCC	TAC	TTT	GAC	TAC
VH 18	GCA	AGA	TACCACCCGGGGATAT	TAC	TTT	TAC	TAC
VH 32	GCA	AGA	TACCACCCGGGGATAT	TAC	TTT	GCC	TAC
VH 68	GCA	CGG	GAATACCCGGGTGGATAC	GGC	TTT	GAC	TAC
VH 91	GCT	CGT	ACCCGGTATAGCTATGGCTATTCCTAC	GGC	TTT	GAC	TAC
VH 83	GCC	CGA	GAGATAACCGGGGGCGGATCTCACTAC	GGC	TTT	GAC	TAC
VH 12	GCA	AGA	GAGGCAGGG	GCT	TTT	GAC	TAC
VH 43	GCA	AGA	GAGTATTGGGGTAATGGT	GCT	TTT	GAC	TAC
VH 85	GCC	CGA	GATTGGGGTAATAGCCACAAT	GCT	TTT	GAC	TAC
VH 53	GCA	AGA	TACGCGGTAGCTGGTATACAT	GCT	TTT	GAC	TAC
VH 76	GCA	CGC	CAGGGTAGCTGGTATTAT	GCT	TTT	GAC	TAC
VH 21	GCA	AGA	TCTCGAGGTTAC	GGC	TTT	GAC	TAC
VH 4	GCA	AGA	TACCGAGGTTAC	GGC	TTT	GAC	TAC
VH 41	GCA	AGA	GATTATAGCTATGGTAGTGACGAC	TAC	TTT	GAC	TAC
VH 14	GCA	AGA	GAGTACGCGGCAGGGTATAAT	TAC	TTT	GAC	TAC
VH 23	GCA	AGA	GCGGGAGAT	TAC	TTT	GAC	TAC
VH 88	GCC	AGA	GCGGGGCCTACGTACGGCGAT	TAC	TTT	GAC	TAC
VH 52	GCA	AGA	GCTCCCGGATGGACT	GCT	TTT	GAC	TAC
VH 7	GCA	AGA	GGGTCCGGATGGACT	GCT	TTT	GAC	TAC
VH 35	GCA	AGA	GGGAGCACCGGT	TAC	TTT	GAC	TAC
VH 36	GCA	AGA	GGGAGCCCGGCCGGT	TAC	TTT	GAC	TAC
VH 34	GCA	AGA	GAACATTATAGCTCCAAT	GCT	TTT	GAC	TAC
VH 17	GCA	AGA	GAATATTATAACTACGACGGT	GCT	TTT	GAC	TAC

Supplementary Fig. 12. Partial nucleotide sequences and V_H-D_H-J_H junction diversity of the VH domain of IgM heavy chain in bighead catfish. Core DH nucleotides are highlighted in yellow. Palindromic sequences or P-nucleotide are underlined with red. The N-diversity or N-nucleotides are non-highlighted letters which essentially observed at the VH-DH-JH junctions. The partial sequences are from a hundred unique nonredundant clones of VH domain, NCBI accession number sets are ordered from MN934442 to MN934541, respectively. Core DH segments were predicted using online tools, IgBLAST (https://www.ncbi.nlm.nih.gov/projects/igblast/), IMGT/HighV-QUEST (http://www.imgt.org/IMGT_vquest/input), VDJsolver 1.0 Server (http://www.cbs.dtu.dk/services/VDJsolver/).

VH Number	VH	DH	JH
VH 6	GCA AAG	TACATAGGGTCTGGAGCC	TAC TTT GCC TAC
VH 6	GCA AAA	CCGCTCGGCTACGCTGAC	TAC TTT GAC TAC
VH 8	GCA AGG	ACCGATGAC	TAC TTT GAC TAC
VH 22	GCA AGC	CCGTGGGGTAGTGGGGAC	TAC TTT GAC TAC
VH 43	GCA AAG	TACAGGGACCATAGCCACCGGTACGAC	TAC TTT GAC TAC
VH 29	GCG CGA	GGTTATACCCGGGTGGGGGACTACGGT	TAC TTT GAC TAC
VH 71	GCC CGT	CGGGTAGTGGGATACTACGT	TAC TTT GAC TAC
VH 31	GCT CGG	GAACCGGTTAGCTATGGTAGTGCTACC	TAC TTT GAC TAC
VH 72	GCA CGC	AGAACGTATAGCTCTGTCTATCACGGT	GCT TTT GAC TAC
VH 75	GCC CGA	GTCGACTATAGCTCTGTCTATTCCTACAAT	GCT TTT GAC TAC
VH 14	GCA AAG	TACCGGGATGGATACGGT	GCT TTT GAC TAC
VH 44	GCA AGA	GACTGGATCGACGGT	GCT TTT GAC TAC
VH 53	GCC CGG	GAGATCAGTCCTGGTGTCACC	TAC TTC GAC TAC
VH 86	GCA AGA	GACCGGGGTAGTGGTGAG	TAC TTC GAC TAC
VH 28	GCA AAG	TTGGATAGCAGCTCG	TCC TTT GAC TAC
VH 94	GCA CGC	TACGGGAGCTGGAGCAGCCGC	TAC TTT GAC TAC
VH 54	GGC AGG	CGAGCTGGAGCTGGC	TAC TTT GAC TAC
VH 67	GCT CGT	CGCATGAGGGATAGCAGCTGGAGT	TAC TTT GAC TAC
VH 100	GCT CGT	TTCCCAGCTGGAGGC	TAC TTC GAC TAC
VH 19	GCA AGC	CTTTATGGTAGCTACGCCGGCTAC	TAC TTT GAC TAC
VH 33	GCG CGC	AACATACATGGTAGC	TAC TTT GAC TAC
VH 76	GCG CGC	AACATCCGTAGA	TAC TTT GAC TAC
VH 19	GCA AGC	CTTTATGGTAGCTACGCCGGCTAC	TAC TTT GAC TAC
VH 33	GCG CGC	AACATACATGGTAGC	TAC TTT GAC TAC
VH 76	GCG CGC	AACATCCGTAGA	TAC TTT GAC TAC
VH 66	GCA AGA	TACGAACGTTACGGGGGG	GCT TTT CAC TAC
VH 90	GCA AGA	CACTATATACCCGGAAC	GGC TTT CAC TAC
VH 63	GCG CGA	GAGAGGCAAGAGGGGCAGCCAC	GGC TTT CAC TAC
VH 73	GCT GTC	TATTACTGCGTTCGCTACATGAGG	GGC TTT CAC TAC
VH 36	GCA AAA	GGACATCCATATAGCTGGAGC	AGC TTT CCC TAC
VH 7	GCA AGA	GTCGGGCTCTACTAC	GGC TTT GAC TAC
VH 18	GCA AGA	GTCGGGCTCTACTAC	GGC TTT GAC TAC
VH 35	GCA AGA	GGCCGGTATAGTGGCTATAACTATGGT	TTC TTT GAC TTC
VH 48	GCA AGA	GGCCGGTATCCTGGCTATCCCTATGGT	TTC TTT GAC TAC
VH 87	GCA AGA	TACAGGTATAGTGGCTATGGTGAGGGT	GCT TTC GAC TAC
VH 84	GCC AGA	GAGTACTCGGGT	CGG TTT GAC TAC
VH 1	GCA AAG	TACATAGGGTCTGGAGCC	TAC TTT GAC TAC

Supplementary Fig. 13. Partial nucleotide sequences and V_H-D_H-J_H junction diversity of the VH domain of IgM heavy chain in north African catfish. Core DH nucleotides are highlighted in yellow. Palindromic sequences or P-nucleotide are underlined with red. The N-diversity or N-nucleotides are non-highlighted letters which essentially observed at the VH-DH-JH junctions. The partial sequences are from a hundred unique nonredundant clones of VH domain, NCBI accession number sets are ordered from MN934542 to MN934641, respectively. Core DH segments were predicted using online tools, IgBLAST (https://www.ncbi.nlm.nih.gov/projects/igblast/), IMGT/HighV-QUEST (http://www.imgt.org/IMGT_vquest/input), VDJsolver 1.0 Server (http://www.cbs.dtu.dk/services/VDJsolver/).

VH Number	VH	DH	JH
VH 21	GCA AGG	TTGTATAACTCTGGCTATGGTGCCGTCTAC	GGC TTT GAC TAC
VH 95	GCG CGA	CAGTCAGATAGCAGCTGGGGTCCC	GGC TTT GAC TAC
VH 67	GCA AGA	GGAGAAAACGAAATCACTACGCCTACC	TAC TTC GAC TAC
VH 71	GCA AGA	TATCCGGGATTACC	TAC TTC GAC TAC
VH 37	GCA AGA	ACGGGGGTGGCC	TAC TTC GAC TAC
VH 94	GCC CGG	GAGATCAGTAGTGGTGTCACC	TAC TTC GAC TAC
VH 22	GCA AAA	TACTATAACTACGCC	TAC TTC GAC TAC
VH 06	GCG GGA	GAGGGGGCTCCTACC	TAC TTC GAC TAC
VH 18	GCC AAA	GGTGGTTATACT	TAC TTC GAC TAC
VH 12	GCA ATG	TATAGTTTTGGC	GTC TTC GAC TAC
VH 24	GTC CGG	GGACTTGACTAC	GTC TTC GAC TAC
VH 34	GCG AGT	ACGGCCCCCGGT	GGT TTT GAC TAC
VH 25	GGA AAA	GGACAATATGGGGGGTCCGAC	TAC TTT GAC TAC
VH 15	GGA AGA	TGCCCCTATTGGGGTAGTAGTGAT	TAC TTT GAT TAC
VH 13	GCA AGA	ACCGCTGGACCAGCTCTC	CCT TTT GAC TAC
VH 39	GCC AGA	CATTTGACGTATCTGGGTCC	GTC TTT GAC TAC
VH 88	GCC CGA	GAGATAACCGGGGGCGGATCTCACTAC	GGC TTT GAC TAC
VH 26	GCA AGA	GGCCGGTATAGTGGCTATAACTATGGTGCC	TTC TTT GAC TTC
VH 85	GCA AGA	GAGTACGCGGCAGGGTATAAT	TAC TTT GAC TAC
VH 43	GCA AGA	GAGTACGCGGCAGGGTATAAT	TAC TTT GAC TAC
VH 42	GCA AGA	GAGTACGCGGCAGGGTATTAT	TAC TTT CAC TAC
VH 07	GCA AGA	GCGGGAGAT	TAC TTT GAC TAC
VH 30	GCA CGA	GAGGGAGAT	TAC TTT GAC TAC
VH 36	GCA GTA	GAGACCCCGGGGTAT	TAC TTT GAC TAC
VH 02	ACC CGA	CTGGCTGGGGGGGATGAT	TAC TTT GAC TAC
VH 01	GCA GAA	AATACGGGTCACGAA	TAC TTT GAC TAC
VH 60	GCA AGA	GGCCGTCGGGAA	TAC TTT GAC TAC
VH 27	GCA AGA	GAACTATGGATCTGGTATTTGA	GAC TTT GGC TAC
VH 11	GCA AGA	GTCGGTATAATGGCGTACGGT	GCT TTT GAC TAC
VH 17	GCA AGA	GTCGGTATAATGGCGTACGGT	GCT TTT GAC TAC
VH 33	GCC AGA	GAGTATAGCTCTGTCTATGGTGCCCTCAAT	GCT TTT GAC TAC
VH 38	GCA AGA	GATACCACTGTATAGCTCTGTCTATGGTGCTACGGT	GCT TTT GAC TAC
VH 98	GCA CGG	GAATACCCGGGTGGATAC	GGC TTT GAC TAC
VH 09	GCG AAA	CCTCTAACTATGCGGTAC	GGC TTT GAC TAC
VH 28	GCA AGA	GAGAGGGACAAT	GCT TTT GAC TAC
VH 86	GCA AGA	GAACATTATAGCTCCAAT	GCT TTT GAC TAC
VH 04	GCA AGA	CTGGGGTAC	GCT TTT GAC TAC
VH 16	GCA AGA	CTCGCTCATTCGGGTAGTGGTTAT	ACT TTT GAC TAC
VH 05	GCA AAA	TTAACAGCTAAT	GCT TTT GAC TAC
VH 80	GCA AGA	TACGCGGTAGCTGGTATACAT	GCT TTT GAC TAC
VH 29	GCA AGA	GACGCAGGTGGAGCAGGGGTG	GTC TTT GAC TAC
VH 44	GCA AGA	TACGAACGTTACGGGGGG	GCT TTT GCC TAC

Supplementary Fig. 14. Partial nucleotide sequences and V_H-D_H-J_H junction diversity of the VH domain of IgM heavy chain in hybrid catfish. Core DH nucleotides are highlighted in yellow. Palindromic sequences or P-nucleotide are underlined with red. The N-diversity or N-nucleotides are non-highlighted letters which essentially observed at the VH-DH-JH junctions. The partial sequences are from a hundred unique nonredundant clones of VH domain, NCBI accession number sets are ordered from MN934642 to MN934741, respectively. Core DH segments were predicted using online tools, IgBLAST (https://www.ncbi.nlm.nih.gov/projects/igblast/), IMGT/HighV-QUEST (http://www.imgt.org/IMGT_vquest/input), VDJsolver 1.0 Server (http://www.cbs.dtu.dk/services/VDJsolver/).

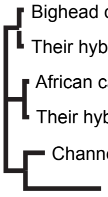

***ighμ*-CH1**		1	2	3	4	5	6
Bighead catfish	**1**	-	100.00	82.35	82.35	72.55	38.61
Their hybrid (mIgMH)	**2**	100.00	-	82.35	82.35	72.55	38.61
African catfish	**3**	82.35	82.35	-	100.00	66.67	38.61
Their hybrid (sIgMH)	**4**	82.35	82.35	100.00	-	66.67	38.61
Channel catfish	**5**	72.55	72.55	66.67	66.67	-	47.52
Zebrafish	**6**	38.61	38.61	38.61	38.61	47.52	-

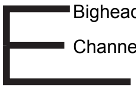

**non-*ighμ* exons**		1	2	3
Bighead catfish	**1**	-	57.73	34.63
Channel catfish (IgDH)	**2**	57.73	-	36.49
Zebrafish (IgDH)	**3**	34.63	36.49	-

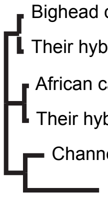

***ighμ*-CH2**		1	2	3	4	5	6
Bighead catfish	**1**	-	100.00	77.23	77.23	57.84	36.89
Their hybrid (mIgMH)	**2**	100.00	-	77.23	77.23	57.84	36.89
African catfish	**3**	77.23	77.23	-	100.00	57.28	33.98
Their hybrid (sIgMH)	**4**	77.23	77.23	100.00	-	57.28	33.98
Channel catfish	**5**	57.84	57.84	57.28	57.28	-	34.62
Zebrafish	**6**	36.89	36.89	33.98	33.98	34.62	-

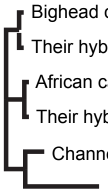

***ighμ*-CH3**		1	2	3	4	5	6
Bighead catfish	**1**	-	100.00	69.70	69.70	45.92	32.99
Their hybrid (mIgMH)	**2**	100.00	-	69.70	69.70	45.92	32.99
African catfish	**3**	69.70	69.70	-	100.00	43.75	25.00
Their hybrid (sIgMH)	**4**	69.70	69.70	100.00	-	43.75	25.00
Channel catfish	**5**	45.92	45.92	43.75	43.75	-	30.85
Zebrafish	**6**	32.99	32.99	25.00	25.00	30.85	-

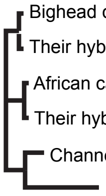

***ighμ*-CH4**		1	2	3	4	5	6
Bighead catfish	**1**	-	100.00	74.40	74.40	54.03	43.22
Their hybrid (mIgMH)	**2**	100.00	-	74.40	74.40	54.03	43.22
African catfish	**3**	74.40	74.40	-	100.00	57.72	44.07
Their hybrid (sIgMH)	**4**	74.40	74.40	100.00	-	57.72	44.07
Channel catfish	**5**	54.03	54.03	57.72	57.72	-	42.62
Zebrafish	**6**	43.22	43.22	44.07	44.07	42.62	-

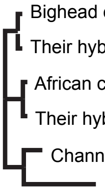

***ighμ*-TM**		1	2	3	4	5	6
Bighead catfish	**1**	-	100.00	97.50	-	84.00	76.00
Their hybrid (mIgMH)	**2**	100.00	-	97.50	-	84.00	76.00
African catfish	**3**	97.50	97.50	-	-	85.71	80.95
Their hybrid (sIgMH)	**4**	-	-	-	-	-	-
Channel catfish	**5**	84.00	85.71	85.71	-	-	72.00
Zebrafish	**6**	76.00	80.95	80.95	-	72.00	-

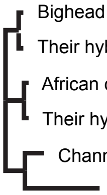

**Overall *ighμ*-CH1-CH2-CH3-CH4**		1	2	3	4	5	6
Bighead catfish	**1**	-	100.00	75.81	75.81	57.48	38.44
Their hybrid (mIgMH)	**2**	100.00	-	75.81	75.81	57.48	38.44
African catfish	**3**	75.81	75.81	-	100.00	56.97	36.34
Their hybrid (sIgMH)	**4**	75.81	75.81	100.00	-	56.97	36.34
Channel catfish	**5**	57.48	57.48	56.97	56.97	-	39.95
Zebrafish	**6**	38.44	38.44	36.34	36.34	39.95	-

Supplementary Table 1. Similarity of amino acid sequences of the *ighμ* gene of the bighead catfish, north African catfish, hybrid catfish, channel catfish and zebrafish. Percent sequence similarity of *ighμ* gene determined using MATGAT version 2.0 software.
